# Flow‐to‐Friction Transition in Simulated Calcite Gouge: Experiments and Microphysical Modeling

**DOI:** 10.1029/2020JB019970

**Published:** 2020-11-18

**Authors:** Jianye Chen, B. A. Verberne, A. R. Niemeijer

**Affiliations:** ^1^ State Key Laboratory of Earthquake Dynamics Institute of Geology, China Earthquake Administration Beijing China; ^2^ HPT Laboratory, Department of Earth Sciences Utrecht University Utrecht The Netherlands; ^3^ Faculty of Civil Engineering and Geosciences Technical University of Delft Delft The Netherlands; ^4^ Geological Survey of Japan National Institute of Advanced Industrial Science and Technology Tsukuba Japan

**Keywords:** calcite friction, flow‐to‐friction transition, brittle‐to‐ductile transition, microphysical model, earthquake nucleation, rock deformation mechanisms

## Abstract

A (micro)physical understanding of the transition from frictional sliding to plastic or viscous flow has long been a challenge for earthquake cycle modeling. We have conducted ring‐shear deformation experiments on layers of simulated calcite fault gouge under conditions close to the frictional‐to‐viscous transition previously established in this material. Constant velocity (*v*) and *v*‐stepping tests were performed, at 550°C, employing slip rates covering almost 6 orders of magnitude (0.001–300 μm/s). Steady‐state sliding transitioned from (strong) *v*‐strengthening, flow‐like behavior to *v*‐weakening, frictional behavior, at an apparent “critical” velocity (*v*_*cr*_) of ~0.1 μm/s. Velocity‐stepping tests using *v* < *v*_*cr*_ showed “semi‐brittle” flow behavior, characterized by high stress sensitivity (“*n*‐value”) and a transient response resembling classical frictional deformation. For *v* ≥ *v*_*cr*_, gouge deformation is localized in a boundary shear band, while for *v* < *v*_*cr*_, the gouge is well‐compacted, displaying a progressively homogeneous structure as the slip rate decreases. Using mechanical data and post‐mortem microstructural observations as a basis, we deduced the controlling shear deformation mechanisms and quantitatively reproduced the steady‐state shear strength‐velocity profile using an existing micromechanical model. The same model also reproduces the observed transient responses to *v*‐steps within both the flow‐like and frictional deformation regimes. We suggest that the flow‐to‐friction transition strongly relies on fault (micro)structure and constitutes a net opening of transient microporosity with increasing shear strain rate at *v* < *v*_*cr*_, under normal stress‐dependent or “semi‐brittle” flow conditions. Our findings shed new insights into the microphysics of earthquake rupture nucleation and dynamic propagation in the brittle‐to‐ductile transition zone.

## Key Points

- We present a transition from flow to friction with increasing slip rate for a simulated carbonate fault sheared at 550°C
- A microphysically based model reproduces the lab‐observed flow‐to‐friction transition, including the transient frictional/flow behaviors
- Faults exhibit semi‐brittle flow by creep cavitation prior to earthquake rupture nucleation at the BDT zone

## 1 Introduction

Within the seismogenic zone and above, fault displacement is achieved by frictional shear deformation, whereas at much deeper levels in the crust, this dominantly occurs by thermally activated creep mechanisms. Under fully “plastic,” “ductile,” or “viscous” conditions, creep flow is fast enough to inhibit unstable fault rupture (Meissner & Strehlau, 1982; Scholz, 1988). The transition with increasing depth (or temperature) from frictional fault slip to fully plastic flow is gradual, involving a competition between time‐insensitive (e.g., granular flow) and thermally activated time‐sensitive (creep) deformation mechanisms over a depth range of several kilometers, or a few tens to hundreds of degrees Celsius (e.g., Bos & Spiers, 2002; Fagereng, 2011; Holdsworth et al., 2001; Imber et al., 2008; Kawamoto & Shimamoto, 1997; Niemeijer & Spiers, 2006; Rowe & Griffith, 2015; Toy et al., 2011). This depth interval, termed the “frictional‐viscous” or “brittle‐to‐ductile transition” (BDT) zone, is characterized by aseismic as well as seismic fault motion, implied by field observations of coexisting mylonites and pseudotachylytes (e.g., Bestmann et al., 2012; Hayman & Lavier, 2014; Stipp et al., 2002; Ueda et al., 2008). A comprehensive understanding of the (micro)physical processes leading to fault rupture is needed to improve numerical models of earthquake fault dynamics within and beyond the BDT (Aharonov & Scholz, 2018, 2019; Jiang & Lapusta, 2016; Shimamoto & Noda, 2014; Tse & Rice, 1986).

To capture the frictional‐viscous or BDT quantitatively and construct or test a constitutive law, a data set on the shear behavior of fault rocks covering a wide range of slip velocities and temperatures is key. Synthetic and natural fault rocks with composite mineralogical compositions (e.g., halite‐ and quartz‐phyllosilicate mixtures) as well as natural fault gouges exhibit transitional shear deformation behavior from frictional slip to viscous flow with decreasing slip rate (e.g., Blanpied et al., 1995; Bos & Spiers, 2002; Chester & Higgs, 1992; Den Hartog et al., 2013; Niemeijer, 2018; Niemeijer et al., 2016; Noda & Shimamoto, 2010; Shimamoto, 1986). To our knowledge, powdered halite remains thus far the only simulated fault rock for which the complete transition from friction to flow with decreasing slip rate has been demonstrated experimentally (Chester, 1988; Shimamoto, 1986). This is important, because laboratory simulations combined with (post‐mortem) microstructural observations enable systematic investigation of the microphysical processes controlling the BDT.

Verberne et al. (2015, 2017) conducted ring‐shear experiments on layers of simulated calcite fault gouge at temperatures (*T*) of 20–600°C and effective normal stresses (*σ*_*n*_) up to 120 MPa. At *σ*_*n*_ of 50 MPa, transitions with increasing temperature were observed from stable (aseismic) *v*‐strengthening to potentially unstable (seismogenic) *v*‐weakening at ~100°C and back to stable *v*‐strengthening at ~600°C. The latter transition, from unstable to stable slip at high temperatures, was interpreted to represent a change from frictional deformation in localized, porous slip zones to (more) distributed, dense ductile flow. Existing constitutive models follow an ad hoc approach, connecting the strength envelops of empirical friction and flow laws (Beeler, 2009; Brace & Kohlstedt, 1980; Chester & Higgs, 1992; Reinen et al., 1992; Shimamoto & Noda, 2014), or else by introducing an empirical *T*‐dependence (Chester, 1994) or an evolution of grain contact area (Aharonov & Scholz, 2018, 2019) to the rate‐ and state‐dependent friction (RSF) laws. However, a fully microphysically based constitutive model, calibrated to (post‐mortem) microstructural observations, is lacking.

We investigate the mechanical and microstructural characteristics of the frictional‐to‐viscous (or brittle‐to‐ductile) transition in simulated calcite gouge, at *T* = 550°C and *σ*_*n*_ = 50 MPa, using displacement rates spanning 6 orders of magnitude. Our aim was to document, for the first time, the complete flow‐to friction transition with increasing slip velocity in simulated fault rock composed of monomineralic calcite. We employed a microphysically based constitutive model for shear of gouge‐filled faults (the Chen‐Niemeijer‐Spiers [CNS] model; Chen & Spiers, 2016; Niemeijer & Spiers, 2007) to quantitatively explain the experimentally observed, steady‐state and transient friction/flow behavior. Specifically, we link fault shear strength to internal changes in porosity with increasing displacement, controlled by the competition between intergranular dilatation by granular flow and creep‐controlled compaction. Using our experimental and microstructural observations as a basis, combined with microphysical modeling, we discuss implications for fault slip behavior within the BDT zone.

## 2 Materials and Methods

### 2.1 Material and Deformation Apparatus

**Figure 1:**
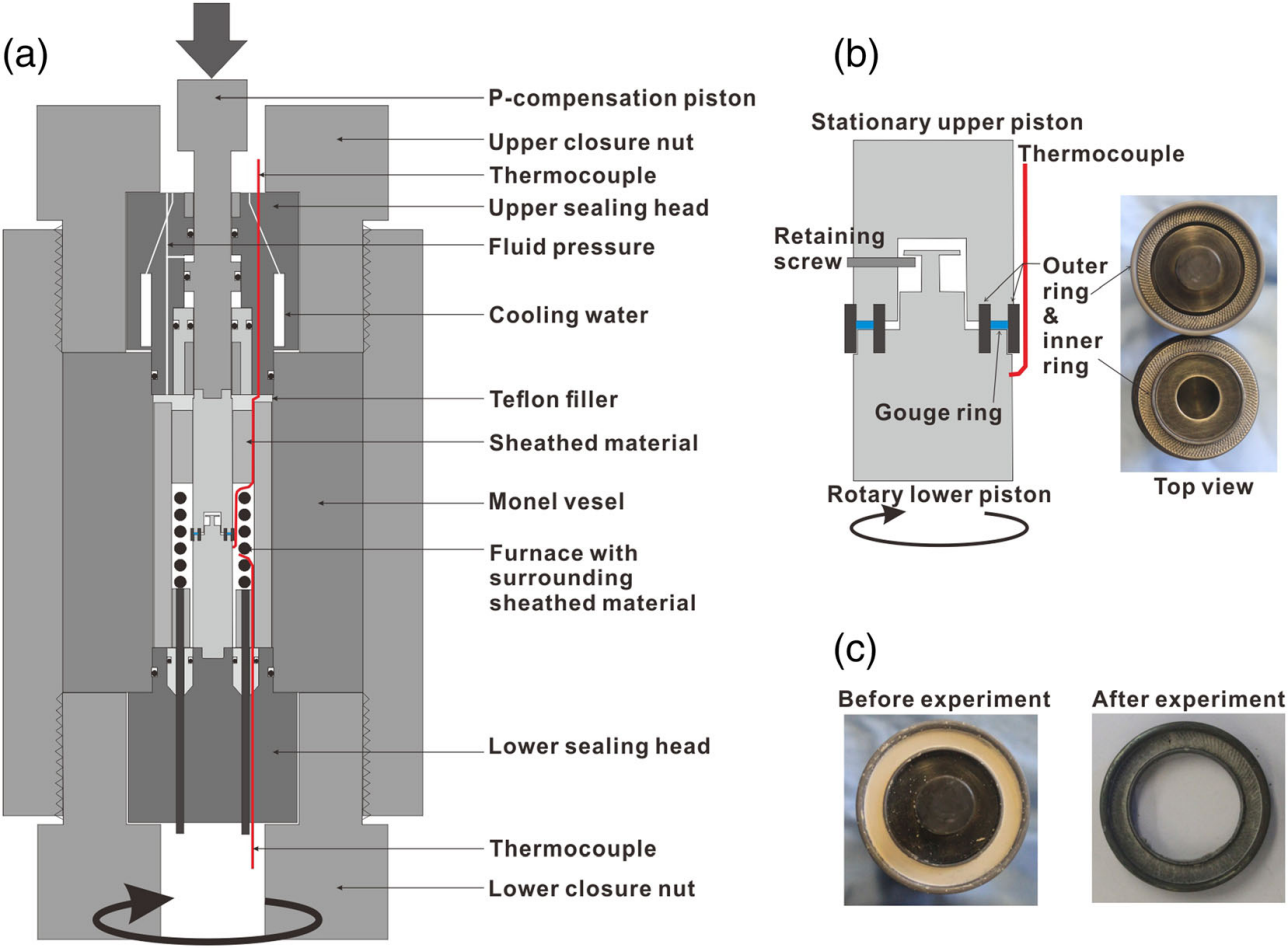
The Utrecht ring‐shear hydrothermal pressure vessel and sample assembly used. (a) Cross section of the pressure vessel, (b) blow‐up of the sample‐piston assembly including the top view of the pair of pistons and confining rings, and (c) simulated gouge layers before and after a shear experiment.

We conducted experiments on simulated fault gouges composed of pure calcite, using the hydrothermal ring‐shear apparatus installed at Utrecht University (Figure 1a). Simulated calcite gouge was prepared from crushed Iceland spar (CaCO_3_) single crystals, sieved to a particle size fraction of less than 28 μm (the same as used by Verberne et al., 2015, 2017). X‐ray diffraction analysis showed the calcite gouge to consist of 98% calcite, with minor (≤ 2%) dolomite. A control experiment was performed on calcite nanopowder, with a nominal starting grain size of <50 nm. In each experiment, ~0.65 g of calcite (nano)powder was distributed in the annular space between two grooved René‐41 Ni‐alloy pistons and confined by an outer and an inner ring with a diameter of 28 and 22 mm, respectively (Figures 1b and 1c). To reduce wall friction, the confining rings were lubricated using Molykote D‐321R anti‐friction coating (an air‐cured dry lubricant). In our experiments, we measured shear displacement using a potentiometer attached to the pressure vessel. Displacement normal to the shearing direction (i.e., compaction/dilatation) was measured using a linear variable differential transducer attached to the Instron frame. For more details on the apparatus, we refer to Niemeijer et al. (2008, 2016).

### 2.2 Experimental Conditions, Procedures, and Data Analysis

All experiments were conducted at a temperature (*T*) of 550°C, an effective normal stress (*σ*_*n*_) of 50 MPa, and a pore fluid pressure (*P*_*f*_) of 100 MPa (the same *T*‐*σ*_*n*_‐*P*_*f*_) conditions as used by Verberne et al. (2017). We used a constant sliding velocity (*v*) ranging between 0.027 and 300 μm/s, or else we employed sequentially stepped values in the range from 0.001 to 300 μm/s. Our experiments achieved total shear displacements (*x*) ranging between 5.4 and 10.2 mm. The test conducted at *v* = 0.027 μm/s ran for ~70.4 hours. Even lower shear displacement rates were achieved by adding an additional gear box to the rotational drive system. This was used in approximately threefold, downward‐only *v*‐stepping tests with an initial *v* of 0.1 μm/s (i.e., *v* = 0.1 → 0.03 → 0.01 → 0.003 → 0.001 μm/s), except one test using 0.1 → 0.03 μm/s. When using an initial sliding velocity of 0.1 μm/s, shear deformation is near‐uniform across the width of the sample layer (see Figure S2), so that subsequently imposed, downward‐only *v‐*steps will avoid shear strain localization. These experiments were extremely time‐consuming and the longest test lasted for 152.5 hours. Finally, we also conducted *v*‐stepping tests covering relatively high slip rates, using threefold and 1.75‐fold steps in the range from 0.1 to 300 μm/s. Table 1 shows a list of all the experiments including the *v*‐stepping sequences imposed where applicable.

**Table 1:** Experiments and Related Key Parameters

Run	*v* (μm/s)	*μ*_*max*_	*x*_*max*_ (mm)	*μ*_*ss*_	Δ*μ*_*r*_	*x*_*ss*_ (mm)	*x*_*final*_ (mm)	*t* (hour)^a^
u513	300	0.99	0.74	0.365	0.007	4.1–7.4	7.4	2.3
u550‐fast^b^	100	1.04	0.52	0.482	0.004	5.0–9.0	10.3	~2.5
u603	30	1.07	1.10	0.588	0.010	5.0–7.3	7.3	2.7
u635	10	1.02	0.95	0.602	0.011	5.0–7.2	7.2	3.5
u550‐slow^b^	10	0.91	0.70	0.548	0.002	4.0–5.2	5.2	~3.5
u594	3.0	0.93	0.80	0.673	0.005	7.5–10.2	10.2	3.5
u507	1.0	0.83	0.55	0.634	0.011	5.0–7.3	7.3	7.0
u593	0.3	0.95	0.60	0.716	0.019	5.0–7.1	7.1	9.5
u516	0.1	1.02	0.90	0.802	0.002	5.0–7.8	7.8	19.8
u508	0.027	0.83	1.50	0.806	0.015	3.5–5.6	5.6	70.4
u499^c^	1.0	0.84	0.45	0.625	0.006	3.5–5.4	10.7	4.0
Continued by	1.0 → 0.54 → 0.3 → 1.0 → 0.30 → 0.1 → 1.0 → 3.0 → 10 → 54 → 100 μm/s							
*μ*_*ss*_	0.625|0.603|0.610|0.601|0.613|0.630|0.613|0.563|0.516|0.476|0.427							
u502^c^	0.1	0.83	1.42	0.761	0.005	5.0–7.2	22.0	27.6
Continued by	0.1 → 0.175 → 0.3 → 0.54 → 1 → 1.75 → 3 → 5.4 → 10 → 17.5 → 30 → 54 → 100 → 175 → 300 μm/s							
*μ*_*ss*_	0.761|0.742|0.715|0.691|0.664|0.644|0.624|0.608|0.588|0.577|0.568|0.554|0.488|0.522|0.520							
u517^c^	0.1	1.02	0.90	0.807	0.015	4.2–7.2	7.8	37.7
Continued by	0.1 → 0.01 μm/s							
*μ*_*ss*_	0.807|0.700							
u597^c^	0.1	1.00	1.60	0.759	0.041	4.0–6.2	8.6	94.6
Continued by	0.1 → 0.03 → 0.01 → 0.003 μm/s							
*μ*_*ss*_	0.759|0.766|0.764|0.549							
u605^d^	0.1	0.90	1.30	0.782	0.004	5.0–6.0	8.5	130.0
Continued by	0.1 → 0.03 → 0.01 → 0.003 → 0.001 μm/s							
*μ*_*ss*_	0.782|0.762|0.672|0.500|0.338							
u604^d^	0.1	0.91	1.15	0.725	0.005	4.0–6.0	8.7	152.5
Continued by	1.1 → 0.03 → 0.01 → 0.003 → 0.001 μm/s							
*μ*_*ss*_	0.725|0.556|0.472|0.399|0.336							

*Note*. *v* = imposed shear velocity, *μ*_*ss*_ = steady‐state friction coefficient, Δ*μ*_*r*_ = standard deviation of the *μ*_*ss*_ measured, *x*_*ss*_ = the shear displacement (*x*) range used to measure *μ*_*ss*_, *μ*_*max*_ = maximum (or apparent yield) friction coefficient, *x*_*max*_ = the *x*‐position to measure the peak friction, *x*_*final*_ = final shear displacement, and *t* = experimental time. All the tests were performed on crushed calcite fault gouge expect a control one (u604) on calcite nanopowder.

^a^ Experimental time (*t*, in hour) before quenching and removing sample from the pressure vessel.

^b^ Results derived from the experiments performed by Verberne et al. (2017) under the same conditions.

^c^ Stable sliding followed by *v*‐steps, for which the uncertainties in *τ*_*ss*_ are displayed in Figure 4.

^d^ Stable sliding at *v* = 0.1 μm/s followed by downward‐only *v*‐steps to 0.001 μm/s, for which the upper bound in *τ*_*ss*_ are estimated by a fitting approach (illustrated in Figure S1, with the fitting parameters given in Table S1).

Upon terminating an experiment, we first removed the shear stress by rotating the vessel including lower internal piston in the opposite direction, at 1 μm/s, followed by a decrease of the normal stress to ~4.2 MPa ( = 1 kN normal load). To prevent vaporization of pore water, we gradually lowered the temperature while simultaneously maintaining the fluid pressure above ~22 MPa (i.e., the supercritical pressure of water; see the represent annealing curve in the supporting information). Upon reaching *T* < 100°C, the vessel was depressurized to atmospheric conditions, the remaining normal load was removed, and the piston‐sample assembly was disassembled. In total, it took about 45 minutes between termination of the experiment and removal of the sample from the pressure vessel.

In the ring‐shear apparatus, the confining rings are unsealed, so the fluid present in the pressure chamber (demineralized water) has direct access to the sample and acts as a pore fluid. The piston‐sample assembly is fluid pressure‐compensated (Figure 1a), so that the effective normal stress (*σ*_*n*_) acting on the sample layer can be calculated directly from the applied normal load, minus a contribution from the O‐ring seals (~2.85 MPa). The externally measured torque was corrected for dynamic seal friction using displacement‐ and pore pressure‐dependent calibrations following Den Hartog et al. (2013). The shear stress (*τ*) supported by the sample was determined assuming a uniform load distribution over the width of the annular sample (3 mm). Standard error propagation analysis showed that δ*τ* ≤ 0.1%. Experiments which employed relatively low displacement rates (*v ≤* 0.1 μm/s) spanning relatively long durations (>20 hours, Table 1) showed fluctuations in *τ* resulting from poor temperature control (± 3°C worst case). The steady‐state shear stress (or shear strength, *τ*_*ss*_) was determined as the average *τ*‐value over a 2‐ to 4‐mm slip interval, with the uncertainty being twice the standard deviation. Individual *v*‐steps in the slow regime (*v* < 0.1 μm/s) did not reach steady state due to the long duration required to achieve the necessary displacement. To circumvent this problem, we estimate the quasi steady state shear stress based on an empirical fit to the experimental data (Figure S1). This should place a maximum bound on the shear strength at these velocities. The friction coefficient (*μ*) was calculated by dividing the shear stress by the seal friction‐corrected *σ*_*n*_‐value, ignoring cohesion of the sample layer (i.e., *μ* = *τ/σ*_*n*_).

### 2.3 Sample Recovery and Microstructural Analysis Methods

For each experiment, recovered sample fragments were impregnated using an epoxy resin, left to harden for several days, and used to prepare polished thin sections in an orientation normal to the shear plane and (sub)parallel to the shear direction. Each sectioned sample was first analyzed using a Leica polarizing light microscope, in transmitted light. Selected sections were subsequently investigated using a FEI Helios Nanolab G3, or a Zeiss Sigma‐0380 scanning electron microscope (SEM). To enable conduction in the SEM, the sectioned samples were sputter‐coated with a ~7 nm thick layer of Pt/Pd. Because our samples are composed almost entirely of calcite, we found that imaging in secondary electron (SE) mode was more effective compared with backscattered electron (BSE) mode. Imaging was achieved with an acceleration voltage of 5 to 10 kV and a beam current of 0.2 to 1.6 nA. Selected SE micrographs were analyzed using the linear intercept method to obtain the grain size (*d*) distribution, assuming *d* = 1.5*L* where *L* is the measured apparent grain diameter as observed in our sectioned samples (following Gifkins, 1970).

To investigate the crystallographic orientation distribution of the calcite grains after shear deformation, we conducted electron backscatter diffraction (EBSD) analysis, using an Oxford Instruments (OI) EBSD detector mounted on the Zeiss Sigma‐0380 SEM. Prior to EBSD measurements, we re‐polished the sections with a silica colloid, followed by coating with a carbon film of less than 4.0‐nm thickness. Automated EBSD mapping of rectangular areas ~25 × 25 μm to 1 × 0.5 mm in size was carried out employing an accelerating voltage of 15 to 20 kV, beam current of ~2 nA, an aperture of 50 μm, a working distance of ~20 mm, and a step size ranging from 0.35 to 2.0 μm depending on the (average) grain size of the mapped area. The Kikuchi band pattern at each measurement or pixel was automatically indexed using OI AZtec software. Indexing in maps of the bulk sample was relatively successful (indexing success rate [ISR] of 50–88%). However, within shear bands, indexing was relatively poor (ISR < 20%), even for the lowest step size employed. For each EBSD map, we carried out repeat measurements in two or three corresponding areas of the sample. Crystallographic orientation data are plotted in upper hemisphere, equal area, and stereographic projections, with contours of mean uniform density (MUD) generated using a half width of 15° and cluster size of 5°.

## 3 Results

### 3.1 Mechanical Data

**Figure 2:**
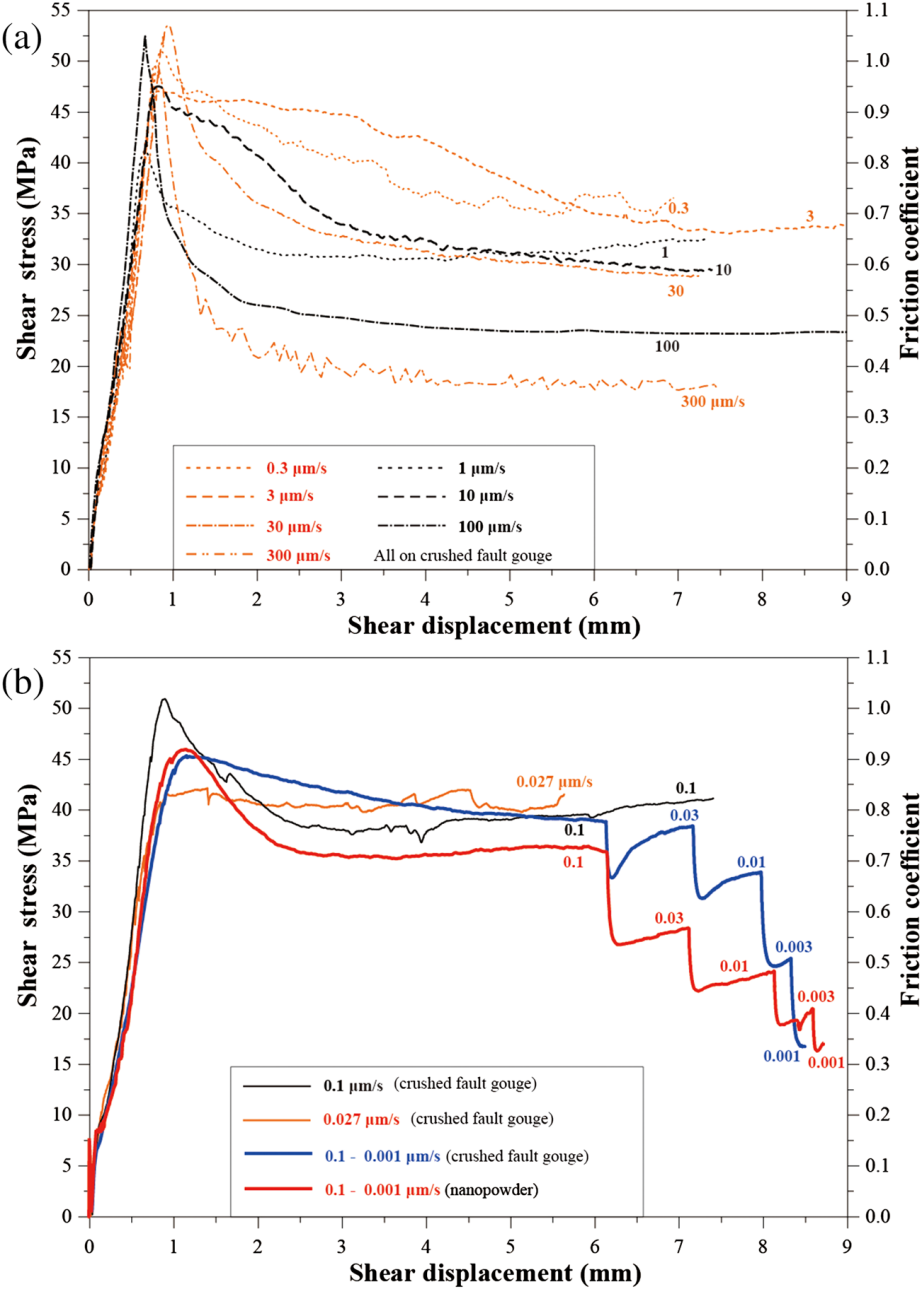
Rotary shear experiments on layers of simulated calcite fault gouge conducted at 550°C, *P*_*f*_ = 100 MPa, and *σ*_*n*_ = 50 MPa, for (a) *v* > 0.1 μm/s and (b) with *v* ≤ 0.1 μm/s. Results from two downward‐only *v*‐stepping experiments are shown in blue and red lines, for the crushed calcite and nanopowder samples, respectively. For clarity, we plot the data to a shear displacement of 9.0 mm.

We plot the shear stress *τ* (or friction coefficient *μ*) versus shear displacement *x* in Figure 2. All experiments and key parameters are listed in Table 1. For each experiment conducted using *v >* 0.1 μm/s, the curves show rapid, near‐linear loading in the first ~0.5 mm of shear displacement and a well‐defined peak friction value of ~1.0 at *x* ≈ 0.5–1.1 mm, followed by rapid, near‐exponential decay to a steady‐state friction value achieved after *x* ≈ 4–5 mm (Figure 2a). By contrast, for experiments using *v* ≤ 0.1 μm/s, initial, near‐linear loading was followed by apparent “yield,” gradual hardening to a maximum friction value, and either gradual weakening or else steady‐state sliding at a near‐constant shear strength value (Figure 2b).

For the *v*‐stepping experiment conducted on crushed calcite using *v* ≤ 0.1 μm/s (u605), the strength values observed at the peak and at steady state during initial sliding at *v* = 0.1 μm/s are broadly consistent with those observed in the constant‐*v* experiment (u516, Figure 2b). Downward steps in *v* consistently triggered a sharp drop in shear resistance, followed by gradual re‐strengthening to a markedly lower, near‐steady‐state strength value (blue line in Figure 2b), implying strong *v*‐strengthening behavior. The test performed on the nanopowder sample, using the same *v*‐stepping sequence, showed similar shear strength‐displacement behavior, but less prominent re‐strengthening following each step (Figure 2b). Furthermore, the steady‐state shear stress or friction at an individual *v*‐step was also lower than that obtained for the crushed calcite at the same velocity.

**Figure 3:**
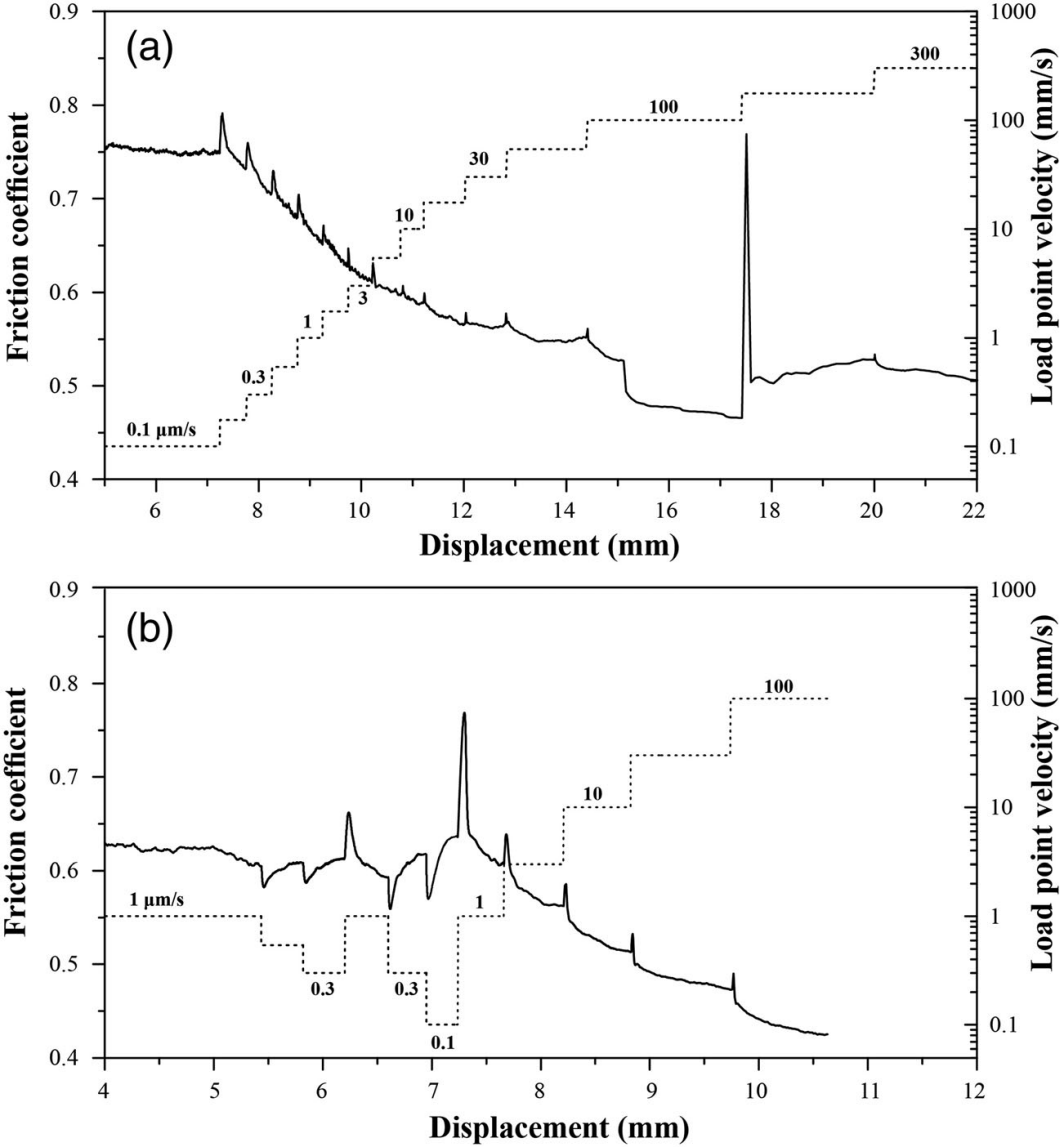
Results from two velocity‐stepping tests in the high velocity range (0.1–100 μm/s). The experimental conditions are the same as that in Figure 2.

In the upward *v*‐stepping tests conducted using *v* > 0.1 μm/s, each individual step showed “classical” RSF behavior, that is, a direct increase in *μ*‐value followed by an exponential decay to a new steady‐state *μ*_*ss*_‐value (Figure 3; for background on RSF theory, see e.g., Marone, 1998). For all the *v*‐steps investigated, *μ*_*ss*_ consistently showed negative rate dependence (i.e., *d* (Δ*μ*_*ss*_)/*d* (ln*v*) < 0), or *v*‐weakening behavior. The “peak” direct effect, in RSF known as the *a*‐value, decreases with increasing *v* (Figures 3a and 3b). At lower velocities, the slip distance required to re‐attain steady‐state sliding (*D*_*c*_ in RSF) is observed to increase, with the *v*‐steps at low displacements not reaching steady state within ~0.5‐mm slip interval. During the interval at *v* = 100 μm/s (experiment u502), sudden, drastic weakening occurred, followed by an extraordinarily large direct effect when stepping to 300 μm/s (Figure 3a). Such drastic weakening was also reported by Verberne et al. (2015), for calcite gouge sheared under the same *T*‐*P*_*f*_‐*σ*_*n*_ conditions, at *v* = 100 μm/s.

**Figure 4:**
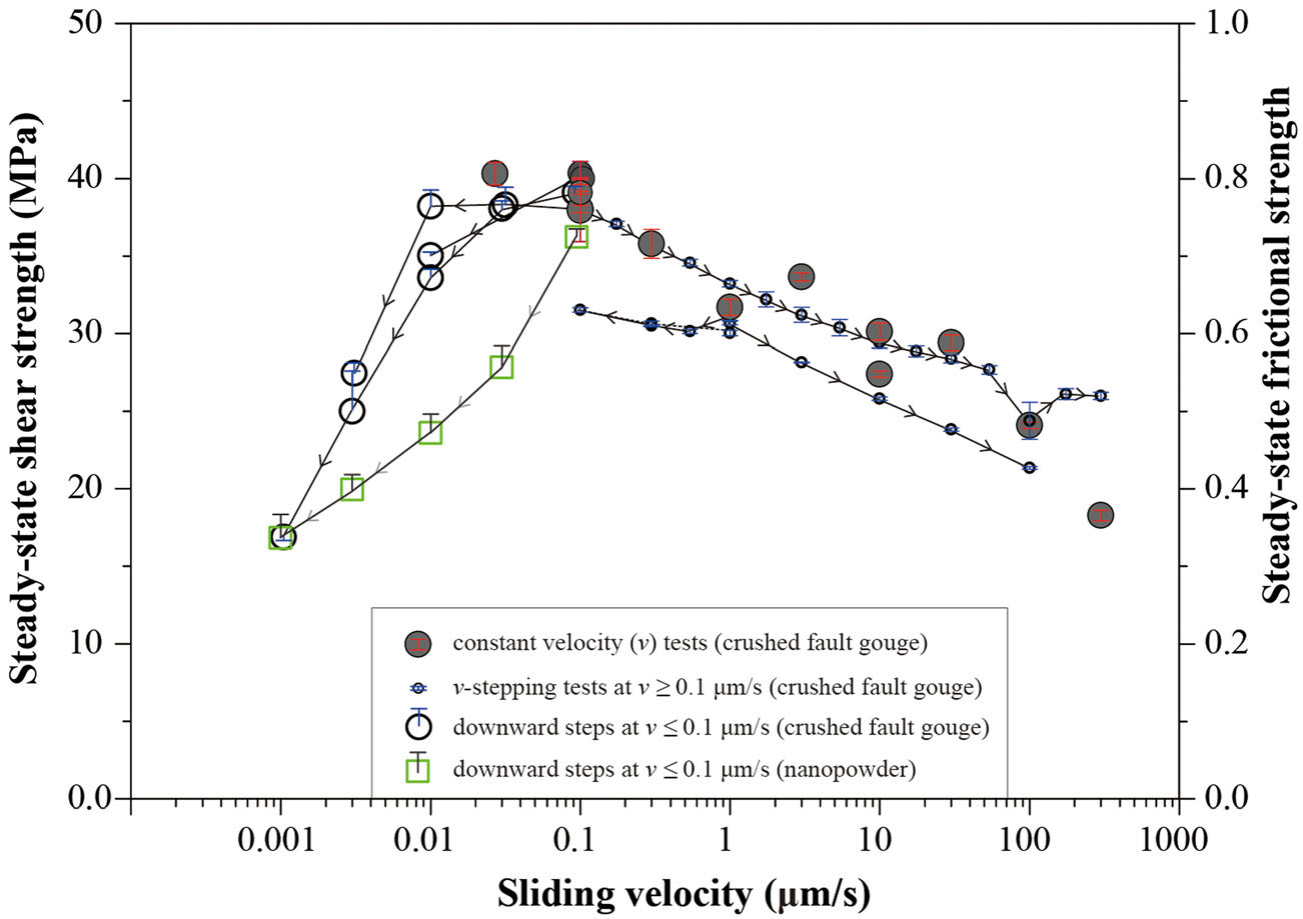
Steady‐state shear strength as a function of sliding velocity for simulated calcite fault gouges sheared at 550°C and 50 MPa effective normal stress conditions. Data are derived from the experiments shown in Figures 2 and 3, plus another two from Verberne et al. (2017) (see Table 1 for details). The error bars give the uncertainties to steady‐state shear strength for the constant‐*v* and upward *v*‐stepping tests. For the downward *v*‐steps (for both crushed calcite and nanopowder samples), we estimated the upper bound using a fitting approach (see Figure S1 and Table S1).

Mean values of the steady‐state shear strength (*τ*_*ss*_) or *μ*_*ss*_ ( = *τ*_*ss*_/*σ*_*n*_) from the constant‐*v* and the *v*‐stepping experiments are plotted against log(*v*) in Figure 4. In the constant‐*v* and upward *v*‐stepping experiments, the uncertainty in the shear strength measurements (± Δ*τ*_*r*_, indicated by the error bars in Figure 4 and given in Table 1) is less than ± 1.3 MPa, except for the data obtained at *v* = 100 μm/s in *v*‐stepping test u502 for which Δ*τ*_*r*_ *=* ± 2.3 MPa. For the downward‐only *v*‐steps, the upper bound of *τ*_*ss*_ obtained from the fitting procedure can be as large as 3.5 MPa higher than the measured level (Table S1). In general, data from all the experiments on crushed calcite are consistent, pointing to a transition with increasing *v* in the sign of d*μ*_*ss*_/dlog(*v*), from positive to negative, around a “critical” velocity (*v*_*cr*_) of ~0.1 μm/s. The nanopowder sample deformed at *v* ≤ 0.1 μm/s also shows a large positive d*μ*_*ss*_/dlog(*v*), but the shear strength is lower than the crushed calcite at the same velocity. Moreover, the two *v*‐stepping experiments using *v* ≥ 0.1 μm/s show consistent slopes for *v* > 1 μm/s (see the small circles, Figure 4), but for *v* ≤ 1 μm/s, the slope in the test using a starting *v* of 1 μm/s is much gentler than the other using 0.1 μm/s (cf. Figures 3a and 3b). We note in this first place that this is because the sample would achieve distinct microstructure after shearing at different starting velocities (i.e., *v* > *v*_*cr*_ vs. *v* ≤ *v*_*cr*_), as explained in the following section.

### 3.2 Microstructures

Upon sample recovery after an experiment, we found that samples that were sheared at low *v* (*v* ≤ 0.1 μm/s) could be extracted as a single, coherent piece, whereas samples sheared at relatively high *v* (>0.1 μm/s) typically broke along shear plane‐parallel and inclined shear fractures, resulting in multiple arc‐shaped fragments. Transmitted light micrographs of sections prepared from each experiment are shown in Figure S2. Below, we describe the microstructures of representative samples u605, u508, and u635, which were deformed using final displacement rates in the experiment (*v*_*final*_) of respectively 0.001, 0.03, and 10 μm/s (see Table 1).

#### 3.2.1 Light and Electron Microscope Observations

**Figure 5:**
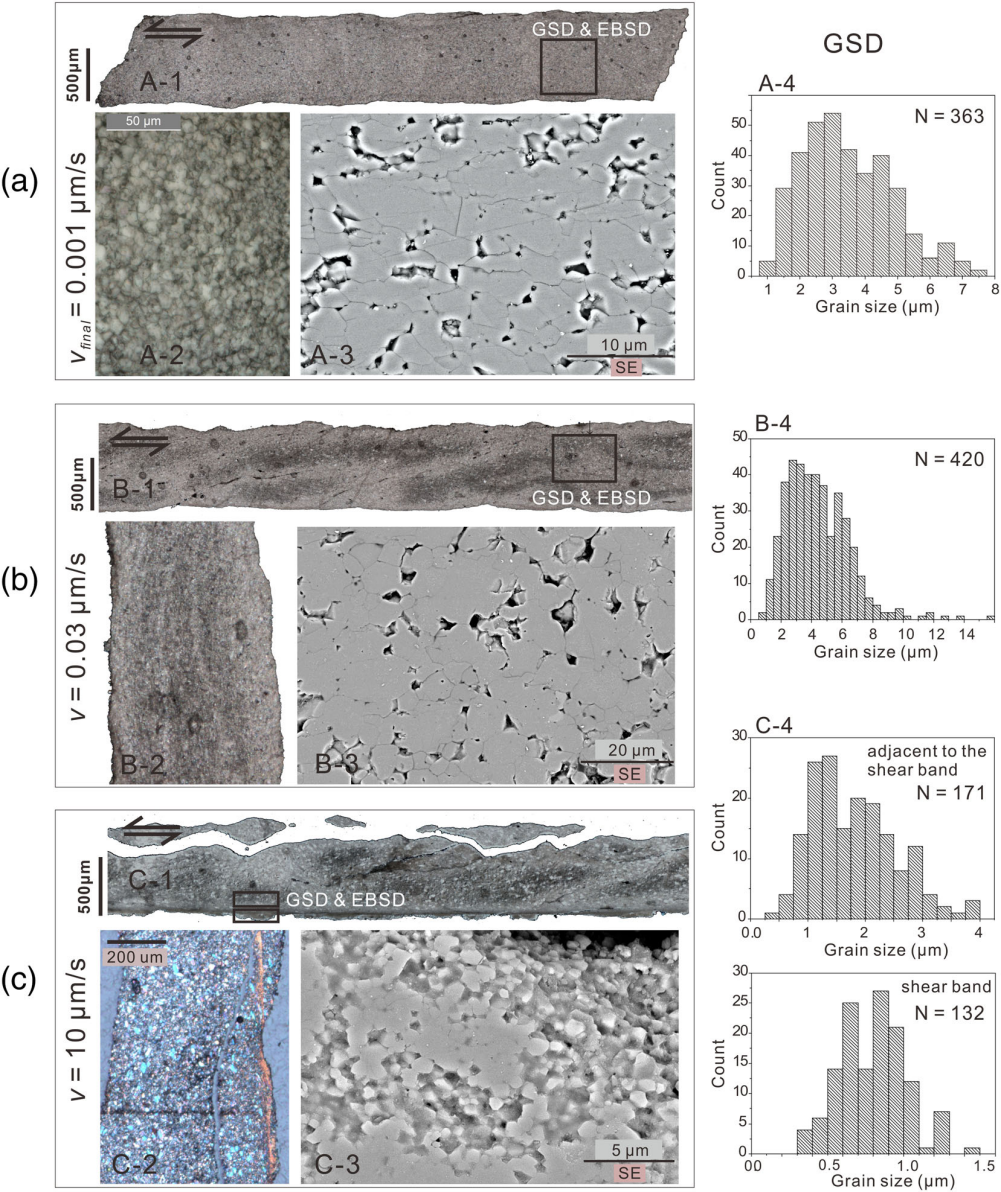
Microstructure of layers of simulated calcite fault gouges from three experiments, sheared at (a) *v*_*final*_ = 0.001 μm/s (u605), (b) constant‐*v* = 0.03 μm/s (u608), and (c) constant‐*v* = 10 μm/s (u635), respectively. Each sample is displayed in four panels: (panels a‐1, b‐1, and c‐1), a transmitted light photomosaic of thin section over the entire gouge layer thickness; (panels a‐2, b‐2, and c‐2), an exaggerated area of potential interests; (panels a‐3, b‐3, and c‐3), a SEM image of a representative area or the shear band if present; and (panels a‐4, b‐4, and c‐4), a histogram of grain size distribution for the selected area. Note that the image shown in panel c‐2 was taken using cross‐polarized light with the gypsum plate inserted. For each sample, imaged‐based grain size distribution analysis was performed on selected areas as marked in rectangles in panels a‐1, b‐1, and c‐1. For the sample sheared at 10 μm/s, these analyses were performed in both the shear band and the adjacent area.

Sample u605 (*v*_*final*_ = 0.001 μm/s) showed a dense, near‐uniform microstructure composed of apparently rounded grains as observed under plane polarized light (PPL) (Figure 5a; see also Figure S2). We observed no evidence for localization of shear deformation. SE micrographs revealed that the sample is characterized by densely packed polygonal grains, frequently with ~120° triple junctions (Figure 5a). Occasionally, the grains are elongated, with a long axis oriented (sub)parallel to the shear plane (Figure 5a). The grain size distribution (GSD) has a range of *d* = 1.0 to ~7.0 μm (*N* = 363) and a mean ( $\bar{d}$) of ~3 μm. For the control experiment on nanopowder (u604), the recovered sample showed a similar near‐uniform microstructures (Figure S2) but a relatively large mean grain size ( $\bar{d}$ = ~10 μm) compared with the starting material ( $\bar{d}$ = 50 nm). Sample u508 (*v*_*final*_ = *v* = 0.03 μm/s) showed light‐ and dark‐gray bands oriented parallel and inclined to the shear plane and direction, as observed using PPL (Figure 5b). We infer that these bands are an artifact from section preparation, possibly representing different degrees of epoxy impregnation. SE micrographs show that this sample has an overall dense microstructure with widespread polygonal grains, resembling the microstructure of sample u605 which was sheared at *v*_*final*_ = 0.001 μm/s (cf. Figure 5a). The GSD (*N* = 420) has a range *d* = 1 to 14 μm and $\bar{d}$ = 4 μm.

Microstructures of samples that were sheared using *v*_*final*_ > 1.0 μm/s consistently showed the presence of a ~20 to 60 μm wide, shear plane parallel zone composed of ultra‐finely comminuted grains, located along at least one of the sample boundaries. For most samples, this boundary (B) shear band was only partially recovered. Light microscope observations of sample u635 (*v*_*final*_ = *v* = 10 μm/s), under crossed‐polarized light (XPL) using the gypsum plate inserted, revealed that the B‐shear is characterized by a strong uniform birefringence and optical extinction, suggestive of a crystallographic preferred orientation (CPO) (Figure 5c). Using a light microscope, grains within the B‐shear cannot be resolved, whereas in the adjacent bulk gouge, the grains are angular, randomly packed, and have a size range close to that of the starting material (*d* = 0.7–50 μm, with $\bar{d}$ = 20 μm, Figure 5c‐1). SE micrographs revealed that the B‐shear is relatively porous for most portions (<3–7%, estimated from the pore area exposed, assuming a circular shape) and that the grains are polygonal to rounded with *d* in the range from 0.3 to 1.5 μm and $\bar{d}$ = 0.8 μm (cf. Figures 5c‐3 and 5a‐3).

#### 3.2.2 EBSD Analyses

**Figure 6:**
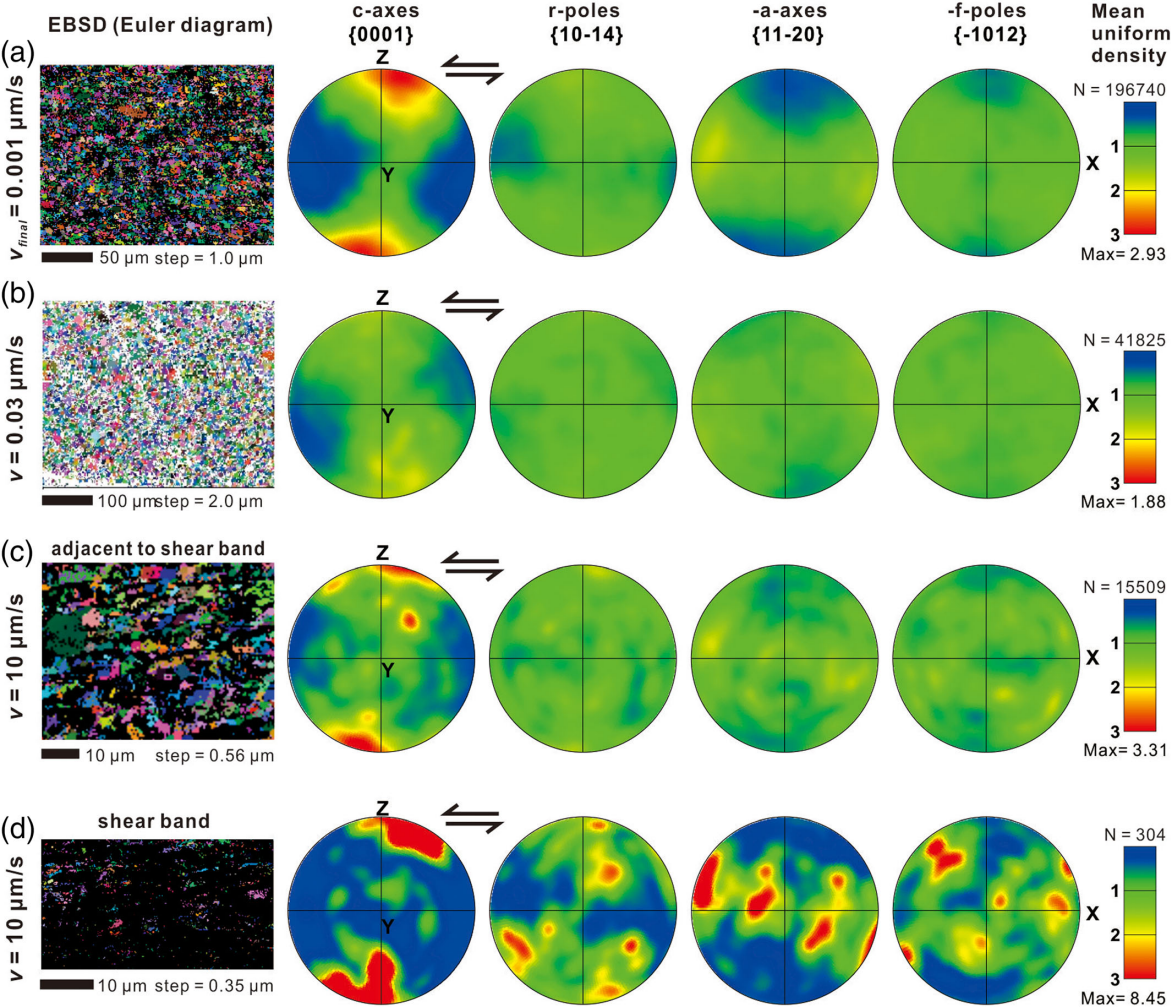
Electron backscatter diffraction (EBSD) of simulated calcite fault gouges retrieved from three experiments, sheared at (a) *v*_*final*_ = 0.001 μm/s (u605), (b) constant‐*v* = 0.03 μm/s (u608), and (c, d) constant‐*v* = 10 μm/s (u635), respectively (see the mapped areas in Figure 5). For sample u635, the analyses were performed in both the shear band and the adjacent area. The left panels give the Euler angle diagram of the mapping area. A step size of 1.0 or 2.0 μm was used in the mapping except for the shear band of u635, where a step size of 0.3–0.6 μm was taken. The EBSD data were plotted in upper hemisphere, equal area pole diagrams for X, Y, and Z directions, respectively.

EBSD mapping was carried out of samples u605, u508, and u635, which were deformed at respectively *v*_*final*_ = 0.001, 0.03, and 10 μm/s (see Table 1). All maps recorded in “slow” experiments u605 and u508 (*v* < 0.1 μm/s) showed ISR ≥ 70% (Figures 6a and 6b; see also results from more areas in Figures S3‐A and S3‐B). By contrast, for maps prepared from sample u635, ISR ≤ 61%, with the lowest value of ~20% for a map of a B‐shear band (Figures 6d and S3‐D). Stereographic projections revealed strong c‐axis maxima in sample u605 (*v*_*final*_ = 0.001 μm/s, Figure 6a) and in the bulk part of sample u635 (*v*_*final*_ = *v* = 10 μm/s, Figure 6c), but less so in sample u508 (*v*_*final*_ = *v* = 0.03 μm/s). For the shear band in sample u635, as evident from the Euler map in Figure 6d, the data are mostly from a few, relatively large grains. Due to poor indexing (ISR ≤ 20%) it remains difficult to compare these and other data obtained from B‐shear bands with other samples.

## 4 Data Analysis and Deformation Mechanisms

### 4.1 Mechanisms Controlling Shear Deformation at *v* < 0.1 μm/s

In view of the high temperature (550°C) used in our experiments and the mechanical and microstructural observations reported above, it is reasonable to suppose that creep processes played at least some role in our experiments, especially at the low displacement rates (*v* < 0.1 μm/s). To investigate this and to identify a suitable constitutive equation that can be used to model our results, we compare the stress sensitivity of the ductile strain rate (the so‐called *n*‐value) as derived from our low‐*v* shear experiments with values determined from compression experiments on dense calcite polycrystals.

**Figure 7:**
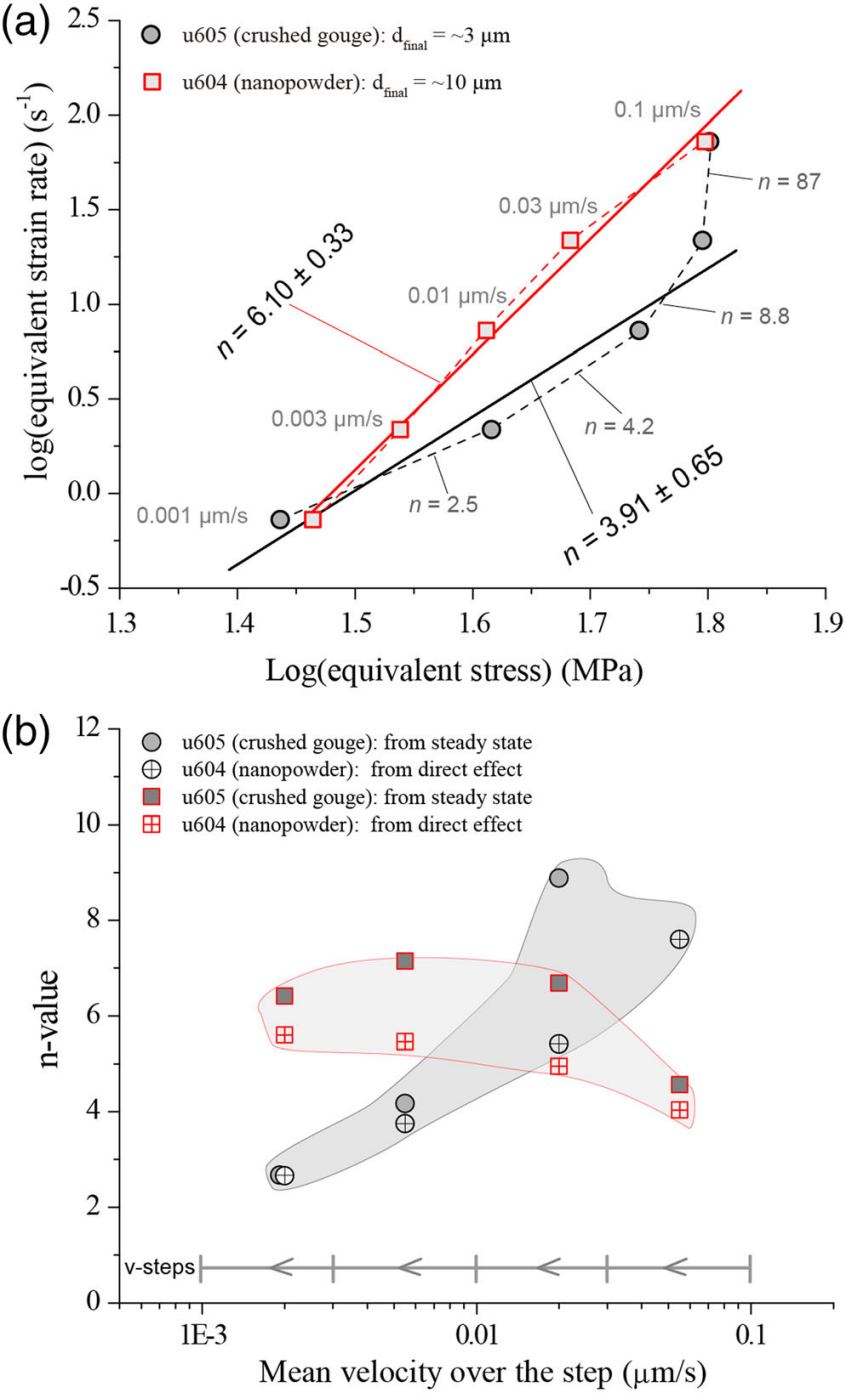
(a) Equivalent strain rate ( $\dot{\varepsilon }$) versus equivalent stress (*σ*) in the logarithmic scale from the two experiments, which were sheared with downward *v*‐stepping sequence from 0.1 to 0.001 μm. Assuming a general creep law of a power law form ( $\dot{\varepsilon }\propto \sigma^{n}$), the *n*‐value can be obtained using the relation *n*  = $\text{dlog}\left(\dot{\varepsilon }\right)/\text{dlog}\left(\sigma \right)\;$ for all the steps, as indicated by the slopes. (b) The *n*‐values as a function of the mean velocity of the *v*‐steps, obtained from both the quasi steady state (shown in panel a) and the direct effect (see the text for explanation).

To this end, we first converted the steady‐state shear stress (*τ*) and shear strain rate ( $\dot{\gamma }$) in our experiments to an equivalent compressive flow (differential) stress (*σ*) and strain rate ( $\dot{\varepsilon }$), using $\dot{\varepsilon }=\dot{\gamma }/\sqrt{3}$ and *σ* $=\sqrt{3}\tau$ (Schmid et al., 1987). The “slowest” experiments on crushed calcite (u605, using *v*_*final*_  =  0.001 μm/s) showed a near‐homogenously deformed microstructure (Figure 5a). Taking a uniform shear zone width *l* of 0.8 mm, this implies that, in experiments using *v*_*final*_ ≤ 0.03 μm/s, $\dot{\gamma }$ ≈ 1.25 × 10^−6^ to 3.75 × 10^−5^ s^−1^and $\dot{\varepsilon }$ ≈ 2.17 × 10^−6^ to 6.50 × 10^−5^ s^−1^. For each *v*‐step interval in the experiment, we calculated $\dot{\gamma }$ and $\dot{\varepsilon }$, assuming constant thickness *W*  =  0.8 mm (Figure 7a). A generalized power law stress dependency of the compressive strain rate (i.e., $\dot{\varepsilon }\propto \sigma^{n}$) implies *n* $=\text{dlog}\left(\dot{\varepsilon }\right)/\text{dlog}\left(\sigma \right)$; hence, an estimate of the *n*‐value can be obtained by taking the slope of the interpolated curve shown in Figure 7a. For each step, the corresponding *n*‐value progressively decreases as *v* decreases (Figure 7b). Ignoring the first step, all values fall in the range from *n* ≈ 2.5 to 8.8, with mean $\bar{n}$  ≈  3.91, which falls between *n*‐values reported for flow of dense calcite polycrystals by diffusion creep (1.1  <  *n* < 1.7) and by dislocation creep (4.2 < *n* < 7.6) (see Table 2; see De Bresser et al., 2002, and references therein). The best match is with the *n*‐value of 3.33 reported by Walker et al. (1990), who best fit a composite, grain size‐ and stress‐dependent flow law to data from compression experiments on synthetic, hot‐pressed calcite aggregates conducted at *σ* < 25 MPa and *T* = 400–700°C. These authors suggested that grain size‐sensitive (diffusion) and grain size‐insensitive (dislocation) creep occurred simultaneously in their experiments.

**Table 2:** Proposed Constitutive Creep Laws of Calcite at High Temperature‐Pressure Conditions

	log*A* (s^−1^ μm^−m^ MPa^−n^)	*E*_*a*_ (kJ/mol)	*n*	*m*	Source
GSS	6.68	213	1.70	3.00	Schmid et al. (1977): regime 3
GSS	7.63	200	1.10	3.26	Herwegh et al. (2003)
GSS + GSI	2.00	190	3.33	1.34	Walker et al. (1990): intermediate *σ/T*
GSI	3.10	420	7.60	‐	Schmid et al. (1980): regime 2
GSI	8.10	428	4.20	‐	Schmid et al. (1980): regime 3
GSI	16.65	584	‐	‐	De Bresser et al. (2002)

*Note*. GSS and GSI denote grain size‐sensitive and grain size‐insensitive creep, respectively. The constitutive creep laws proposed are either in a power form $\dot{\varepsilon }=\text{Aexp}\left(-\frac{E_{a}}{\mathrm{RT}}\right)\frac{\sigma^{n}}{d^{m}}$ or an exponential from $\dot{\varepsilon }=\text{Aexp}\left(-\frac{E_{a}}{\mathrm{RT}}\right)\;\exp\left(\frac{\sigma }{B}\right)$, where *T*, *σ*, and *d* are in units of K, MPa, and μm, respectively. The factor *B* is 2.43 MPa in the exponential law proposed by De Bresser et al. (2002).

As addressed earlier, the shear stress did not reach true steady state due to limited shear displacement, which caused large uncertainty in the *σ*‐value and thus *n*‐value. An alternative approach to derive the stress exponent is from the so‐called direct effect, assuming constant microstructure (e.g.,$Hansen et al., 2012). Taking a large stiffness, the direct effect parameter (i.e., *a*‐value) can be determined using the relation *a* = Δ*μ*_*pk*_/Δ(ln*v*), where Δ*μ*_*pk*_ = *μ*_*pk*_ − *μ*_*pre*_ is the difference between the “peak” and pre‐*v*‐step friction values and Δ(ln*v*) is the logarithm of the size of the *v*‐step. In the case of power law creep, the *a*‐value can theoretically be expressed as *a* = *μ*/*n*. Combining these two relations yields $n=\bar{\mu }\Delta \left(\ln v\right)/\Delta \mu_{\mathrm{pk}}$, where $\bar{\mu }=\left(\mu_{\mathrm{pk}}+\mu_{\mathrm{pre}}\right)/2$ is the mean friction over the direct response. As shown in Figure 7b, the *n*‐values determined from the direct responses are generally consistent with those obtained using quasi steady state friction values, both showing a decrease with decreasing sliding velocity. For the slowest steps (*v* ≤ 0.01 μm/s), the *n*‐values obtained from the two methods fall between 2.5 and 4.0.

Based on the above, we posit that shear deformation at *v* < 0.1 μm/s in our experiments on crushed calcite occurred by a combination of diffusion and dislocation creep processes. Importantly, the operation of dislocation and diffusion creep is consistent with microstructural observations. First, samples sheared at *v* < 0.1 μm/s showed distributed shear deformation, a relatively low porosity (<~2%), and polygonal grains characterized by straight grain boundaries and high‐angle triple junctions (Figures 5a‐3 and 5b‐3). The latter are consistent with microstructures formed in compression experiments on dense calcite polycrystals, which deformed by grain size sensitive creep (Schmid et al., 1977; Walker et al., 1990). Furthermore, the presence of 4–9 μm sized elongated grains in sample u605 (*v*_*final*_ = 0.001 μm/s; Figure 5a) and the c‐axis maximum (Figures 6a and 6c) are suggestive of intracrystalline plasticity (cf. Lafrance et al., 1994; Rutter et al., 1994; Schmid et al., 1987; Walker et al., 1990). Lastly, the GSD measured in samples sheared at *v*_*final*_ < 0.1 μm/s is much narrower than compared with that in the starting material (ranging 1–9 vs. 0.7–50 μm), implying that dynamic and/or static recrystallization played a role in the experiment (Drury et al., 1985). A simple calculation using the equation given by Covey‐Crump (1997) for fluid‐assisted grain growth in dense calcite aggregates with *d* < 10 μm indicates that in our “slow” experiments using *v* ≤ 0.1 μm/s, grain growth is only expected in the first few hours (<10^4^ s) of the experiments. Therefore, this process did not affect our steady‐state data.

Combining all of the above, our interpretation is that shear strain accommodation at *v* < 0.1 μm/s in our experiments on crushed calcite occurred by a combination of diffusion and dislocation creep (hereafter referred to the flow regime). However, around the critical velocity *v*_*cr*_, shear strain accommodation is characterized by a “brittle” component, as indicated by the large stress exponent (*n* ~87, Figure 7a) for *v* = 0.03–0.1 μm/s (Figure 4; Brantut et al., 2013; Chen et al., 2020) and by the “friction‐like” transient response to a step in *v* (Figure 2b; Chester, 1988; Noda & Shimamoto, 2010).

For the nanopowder experiment (u604), the equivalent $\dot{\varepsilon }-\sigma$ data from the quasi steady state show a more or less linear trend in the log‐log space, with a slope indicating an average *n*‐value of 6.10 (Figure 7a). Similar *n*‐values are also obtained from the direct effect (Figure 7b). According to the existing creep laws for calcite at high temperature‐pressure conditions (Table 2), an *n*‐value of ~6 suggests the operation of dislocation creep, which is supported by the fact that large grain size ( $\bar{d}$ = 10 μm) and CPO were observed after the experiment. Remarkably, the nanopowder sample has undergone more (or faster) grain growth (from 50 nm to 10 μm) than the crushed calcite (from ~1.5 to ~3 μm). To better understand this, a microstructural comparison is needed between experiments stopped at varying shear displacements, and using a quenching technique, which is beyond the scope of this study. In the following, we will focus on the crushed calcite samples.

### 4.2 Mechanisms Controlling Shear Deformation at *v* > 0.1 μm/s

All experiments which explored *v* > 1.0 μm/s showed *v*‐weakening behavior (Figure 4). As mentioned above, in these “fast” experiments, the transient response strongly resembled “classical” RSF behavior, and recovered sample fragments consistently showed evidence for shear strain localization in a narrow (20–60 μm), boundary‐parallel (B) shear band (Figures 5c and S2). The presence of a B‐shear suggests that this accommodated the bulk of the imposed shear deformation (Takahashi et al., 2017; Verberne et al., 2017). Assuming a constant, average shear band thickness of ~40 μm, the internal shear strain rate measured ~2.5 × 10^−2^ to 6 s^−1^ for *v* = 1–300 μm/s, which is ~6 orders of magnitude higher than that in experiments conducted using *v* ≤ 0.1 μm/s.

The shear band consists of polygonal or rounded grains, resembling the grain cavitated arrays reported to have formed by Verberne et al. (2017) in experiments conducted under similar *T‐σ*_*n*_ *‐P*_*f*_ conditions (Figure 5c). This, combined with the relatively high shear strain rates acting within the shear bands, implies that granular flow must have played a role. However, plastic creep mechanisms likely also played some role. In view of the high temperatures in our fluid‐saturated experiments (550°C) and small mean grain size in the B‐shear bands compared with samples sheared at *v* < 0.1 μm/s, water‐assisted diffusion creep ( $\dot{\varepsilon }$ ∝ *d* ^−3^) is an obvious candidate. On the other hand, the presence of a CPO, as evident from uniform optical birefringence under a light microscope (Figure 5c‐2), is suggestive of dislocation creep. A c‐axis maximum, similar to the one observed in the low‐*v* experiments, was identified in grains adjacent to a B‐shear (Figures 6c and S3), consistent with that reported by Verberne et al. (2017) for internal shear band grains.

Combining all of the above, our interpretation is that in the flow regime (*v* < 0.1 μm/s), a combination of diffusion and dislocation creep played the dominant role, while at high slip rates (*v* > 0.1 μm/s, hereafter referred to as the friction regime), granular flow played an important role alongside plastic creep process.

## 5 Microphysical Modeling

In this section, we use a previously developed microphysical model for shear of granular media, the CNS model, to simulate the mechanical behavior of calcite gouge observed in our experiments. The CNS model is capable of quantitatively reproducing steady‐state and transient shear behavior, using physics‐based input parameters derived from laboratory observations (Chen & Spiers, 2016). The model assumes that (1) gouge deformation is accommodated by the parallel operation of granular flow and a general “plastic” creep process; (2) granular flow causes gouge dilatation and creep in the normal fault direction leads to compaction, with their competition controlling the evolution of porosity and thus state of the gouge during deformation; and (3) as a result, frictional or flow behavior becomes dominant as velocity, temperature, or normal stress changes, depending on the relative contribution of the two processes. A lower velocity, higher temperature, or higher normal stress causes faster creep and lower porosity, eventually leading to a friction‐to‐flow transition (Chen & Niemeijer, 2017).

Constitutive equations used in this study are the same as the original model (Chen & Spiers, 2016), except that here we use a power law for the plastic creep process (the original assumes pressure solution). Power law creep has also been adopted in our recent study of frictional healing (Chen et al., 2020). All the equations are explained in the supporting information (Equations S1–S6). For details on model development and implementation, we refer to Chen and Niemeijer (2017), Chen and Spiers (2016), Chen et al. (2017), and Niemeijer and Spiers (2007).

### 5.1 Model Framework and Parameters

#### 5.1.1 Governing Equations

In the model, the sheared gouge layer is modeled analogous to a spring‐slider system, composed of a linear spring of stiffness *K* that is activated at a load point at velocity *v*_*imp*_, assuming no inertia:

(1a) $$\dot{\tau }=K\left(v_{\mathrm{imp}}-v\right)$$

In section 4, we showed that, within the range of sliding velocities corresponding with the frictional regime (*v* > 0.1 μm/s) in our experiments, shear plane‐parallel deformation of a gouge layer of thickness *W* occurs by the simultaneous operation of granular flow ( ${\dot{\gamma }}_{\mathrm{gr}}$) and intergranular plastic creep ( ${\dot{\gamma }}_{\mathrm{pl}}$). In the assumed model geometry, granular flow operates in a shear band of width *W*_*sb*_, while intergranular creep may occur involving the entire gouge, including the shear band as well as the adjacent bulk layer (*W*_*bulk*_) (see Text S1). The implication is that

(1b) $$v=W_{\mathrm{sb}}{\dot{\gamma }}_{\mathrm{gr}}+W_{\mathrm{sb}}{\dot{\gamma }}_{\mathrm{pl}}^{\mathrm{sb}}+W_{\text{bulk}}{\dot{\gamma }}_{\mathrm{pl}}^{\text{bulk}}$$

where *W*_*sb*_ + *W*_*bulk*_ = *W* and ${\dot{\gamma }}_{\mathrm{pl}}^{\mathrm{sb}}$ and ${\dot{\gamma }}_{\mathrm{pl}}^{\text{bulk}}$ are the creep strain rates within respectively the shear band and the bulk layer, in the shear direction. For *v* < 0.1 μm/s, shear deformation is more homogeneous; hence, *W* ≈ *W*_*sb*_ and $v=W{\dot{\gamma }}_{\mathrm{gr}}+W{\dot{\gamma }}_{\mathrm{pl}}$.

The state equation governing the evolution of porosity (*φ*) is written as follows:

(2) $$\frac{\dot{\varphi }}{\left(1-\varphi \right)}=\text{tanψ}{\dot{\gamma }}_{\mathrm{gr}}-{\dot{\varepsilon }}_{\mathrm{pl}}^{\mathrm{sb}}$$

Here *ψ*, the average dilatation angle, is expressed as a function of porosity, *tanψ* = 2*H*(*φ*_*c*_ − *φ*), where *H* is a geometrical constant and *φ*_*c*_ the critical porosity for granular flow (Niemeijer & Spiers, 2007). In the presence of slip localization, the evolution of bulk porosity is not considered due to the limited contribution to deformation. Accordingly, *φ* in Equation 2 represents either the uniform porosity at *v* ≤ 0.1 μm/s or the shear band porosity at *v* > 0.1 μm/s.

Equations 1 and 2 are the two governing ordinary differential equations (ODEs) that specify the rate of change in shear stress ( $\dot{\tau }$) and porosity ( $\dot{\varphi }$). In the framework of the CNS model, granular flow strain rate ( ${\dot{\gamma }}_{\mathrm{gr}}$) can be expressed as a function of *τ* and *φ*, while the creep strain rates ( ${\dot{\gamma }}_{\mathrm{pl}}^{\mathrm{sb}}\;$, ${\dot{\gamma }}_{\mathrm{pl}}^{\text{bulk}}$, and ${\dot{\varepsilon }}_{\mathrm{pl}}^{\mathrm{sb}}$) can be calculated from the modified laws for the identified creep mechanism, which are also functions of *τ* and *φ* (Text S1). As in the previous study (Chen & Spiers, 2016), the ODEs were solved using the finite element package COMSOL.

#### 5.1.2 Parameters Used in the Modeling

All parameters and values used in our simulations are listed in Table 3. These parameters are from either the experimental conditions, the microstructure, or the identified creep laws.

1. The temperature and effective normal stress used followed the experimental conditions employed (i.e., *T =* 550°C, *σ*_*n*_ = 50 MPa).
2. Layer thicknesses (*W*), grain size (*d*), and (initial) porosities (*φ*) were set in accordance with post‐mortem microstructural observations, where relevant of the shear band and the bulk sample layer. To simulate flow behavior at low velocities (*v* < 0.1 μm/s), we assumed a homogeneous shear zone of *W* = 800 μm, with *d* ≈ 2–3 μm. Conversely, at high velocities (*v* > 0.1 μm/s), we assumed *W*_*sb*_ ≈ 20–100 μm, *W*_*bulk*_ = 800 − *W*_*sb*_ (μm), and a grain size of respectively 0.8 and 5.0 μm. To match the overall shear strength level observed in our experiments (Figure 4), we assumed a reference grain boundary friction value ${\tilde{\mu }}^{*}$ of 0.43 at *v* = 0.1 μm/s and a rate‐dependent coefficient ( $a_{\tilde{\mu }}$) of 0.01 (Chen & Spiers, 2016). We assumed a critical porosity *φ*_*c*_ of 40% (see Vermeer & De Borst, 1984) and a non‐zero limit porosity *φ*_0_ of 2% (see Text S1 for details).
3. We used a flow stress‐sensitive (*σ*) and grain size‐sensitive (*d*) constitutive law to quantify the creep strain rate ( $\dot{\varepsilon }$), as calibrated to data from compression tests on dense calcite polycrystals by Walker et al. (1990) (see Table 2):

(3) $$\dot{\varepsilon }=\text{Aexp}\left(-\frac{E_{a}}{\mathrm{RT}}\right)\frac{\sigma^{n}}{d^{m}}$$

Here *A* is a pre‐exponential constant (log*A*  =  6.68 s^ − 1^μm^−m^ MPa^−n^), *E*_*a*_ is the activation energy (190 kJ mol^−1^), *T* is the temperature, *R* is the gas constant (8.31 J mol^ − 1^ K^−1^), and *m* = 1.33 and *n* =  3.33 are empirical constants. In the CNS model, we used this creep law for both normal and shear deformation, with slightly different pre‐exponential constants (*A*_*n*_  =  *A* and *A*_*t*_ $={\sqrt{3}}^{n+1}$ *A*, where *A*_*n*_ and *A*_*t*_ are the constants for normal and shear components, respectively; see Text S1 for detailed description). A porosity function is used to account for changing porosity in the frictional regime (Niemeijer & Spiers, 2007).

4. To simulate the transient behavior, the initial shear stress and porosity (either the uniform porosity or the porosity in the shear band) were set at the steady state corresponding to the load point velocity. In the case of slip localization (*v* > 0.1 μm/s), the bulk porosity is set to be 20%.

**Table 3:** Microphysical Model Parameters and Values

Symbol	Description (unit)	Values	Source
*σ*_*n*_	Effective normal stress	50 MPa	Present experiment
*T*	Temperature	550°C	Present experiment
*K*	Stiffness of a simulated fault	6 × 10^11^ Pa/m	This study
*W*	Thickness of the homogeneous gouge layer	0.8 mm	Microstructure
*d*	Nominal grain size of a homogeneous gouge layer	3 (2–4) μm	Microstructure
*W*_*sb*_	Shear band thickness in the case of localized slip	50 (30–100) μm	Microstructure
*W*_*bulk*_	Thickness of the bulk zone in the case of localized slip	0.8 mm *W*_*sb*_	Microstructure
*d*_*sb*_	Nominal grain size of the shear band	0.8 μm	Microstructure
*d*_*bulk*_	Grain size in the bulk layer	5.0 μm	Microstructure
*φ*_*c*_	Critical state porosity for granular flow	0.4	This study
*φ*_0_	Terminal porosity of a compacted gouge	0.02	Chen and Niemeijer (2017)
*φ*_*ini*_	Initial porosity in both shear and bulk layer	0.10	This study
*p*	Sensitivity parameter in porosity function	2.0	Spiers et al. (2004)
*H*	Geometrical parameter for grain package	0.57	Chen and Spiers (2016)
$\tilde{\mu }$ ^*^	Grain boundary (gb) friction coefficient at 1 μm/s	0.45	This study
$a_{\tilde{\mu }}$	Logarithmic rate dependence of gb friction	0.01	Chen and Niemeijer (2017)

*Note*. Values in the brackets give the variations for parametric analysis. Constant parameter values in the creep law are given in Table 2 (GSS + GSI, Walker et al., 1990).

### 5.2 Simulation Results and Comparison With Experiments

#### 5.2.1 Steady‐State Behavior

**Figure 8:**
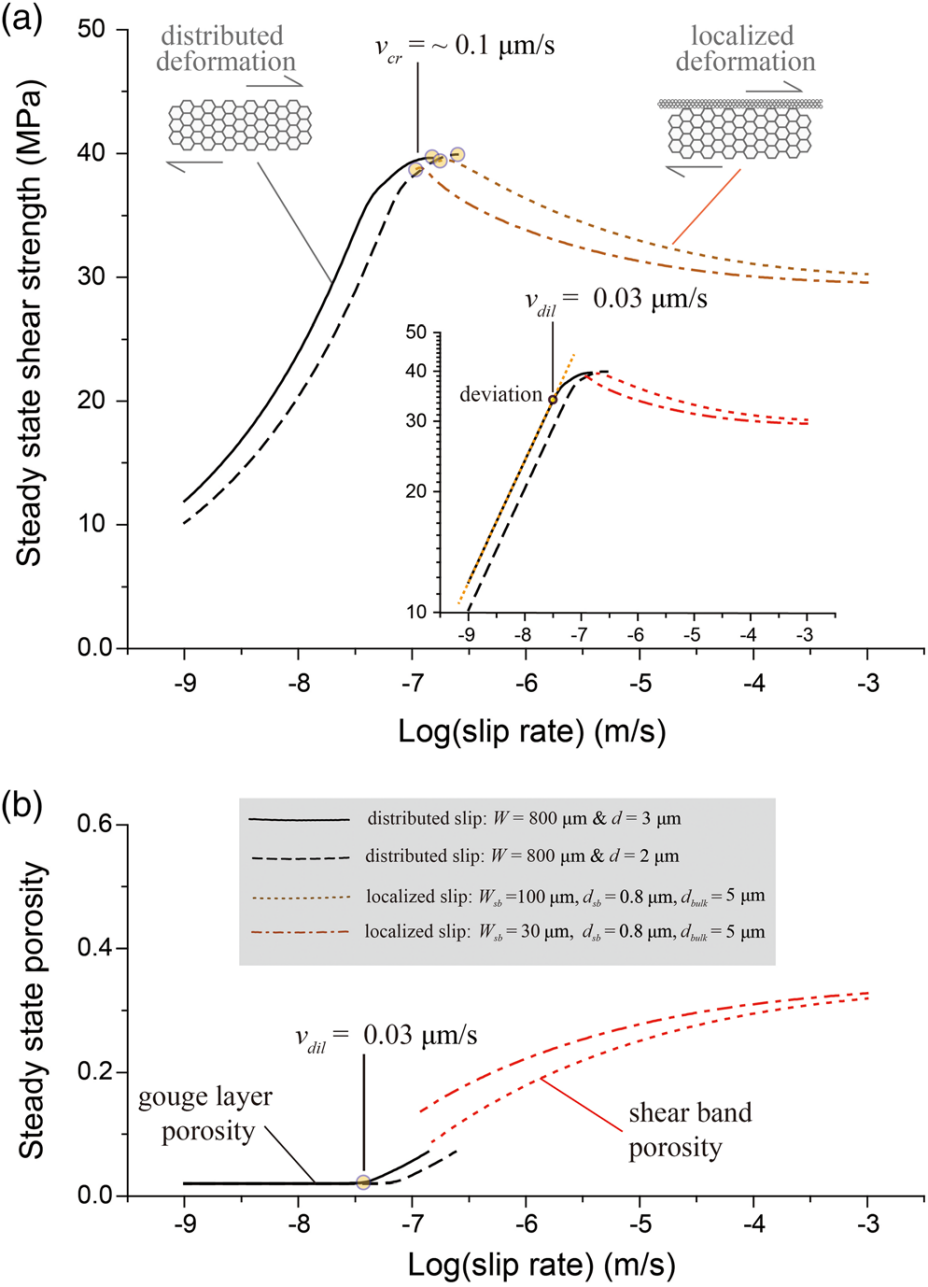
Steady‐state (a) shear strength and (b) porosity as a function of slip velocity for a simulated calcite gouge layer at 550°C and 50 MPa effective normal stress, predicted by the CNS model. The model conditions were set according to the experiments, with different model geometries resembling the microstructures observed at different slip rates. Specifically, for slow slip rates, a uniform gouge layer was assumed, while at high slip rates, we assumed localized slip, with different grain sizes and thicknesses for the shear band and the bulk layer. The predicted results indicate in transition from flow to friction at a critical velocity (*v*_*cr*_) of 0.1 μm, consistent with the observation. The inset graph of (a) shows the same results but in the log‐log scale, where the deviation from a linear line occurs at a velocity corresponding to the onset of dilatation (*v*_*dil*_).

The CNS model output simulating the steady‐state shear strength and porosity change with increasing displacement in our experiments is shown in Figures 8 and S5. We also carried out sensitivity analysis for grain size and shear band thickness. For a homogeneously shearing gouge layer at *v* gouge 1 μm/s, the model predicts strong *v*‐strengthening behavior (Figure 8a), reaching a “background” (or limit) porosity *φ*_0_ (Figure 8b). When plotted in log‐log space (Figure 8a, inset), the *τ‐v* curves are straight lines with dlog(*v*)/dlog(*τ*) = *n* = 3.3 (see Equation 2). As *v* increases, the steady‐state porosity begins to increase from the background value, at the dilatation velocity *v*_*dil*_ = ~0.03 μm/s (Figure 8). This onset of dilatation, or *φ(v)* > *φ*_0_, is associated with a deviation of the *τ‐v* curve from linearity (Figure 8a, inset), implying a higher stress sensitivity (or larger “apparent” *n*‐value). For *v* > *v*_*cr*_ = 0.1 μm/s, constituting localized shear, the model predicts persistent *v*‐weakening and an increasing steady‐state porosity with increasing *v*, with slopes that decrease with increasing *v* (Figure 8). For each shear deformation regime (*v* < *v*_*cr*_ and *v* > *v*_*cr*_), the model outcome is generally consistent with the *τ‐v* profile observed in the experiments (cf. Figures 8 and 4; see a detailed comparison in Figure S4).

Regardless of the grain size or shear band width used, the *τ‐v* curves show a smooth connection between both shear deformation regimes, that is, within a peak shear stress and velocity window of 38 to 40 MPa and 0.1 to 0.25 μm/s (Figure 8). However, there is a relatively large offset in porosity, which is unsurprising since the model assumes a different internal fault structure or geometry for the flow (*v* < *v*_*cr*_) versus the frictional (*v* > *v*_*cr*_) regimes. The microphysical processes controlling the change from distributed to localized slip, at *v* ~ *v*_*cr*_, is not captured by the present model. We note, however, that in the case that there would be no microstructural change at *v* = *v*_*cr*_, the model predicts a continuous transition with increasing slip rate from *v‐*strengthening to *v*‐weakening behavior (Figure S6). This suggests that a flow‐to‐friction transition with increasing slip rate will always emerge from the model and that the microstructure controls the velocity at which the transition from *v*‐strengthening to *v*‐weakening occurs (i.e., the value of *v*_*cr*_).

**Figure 9:**
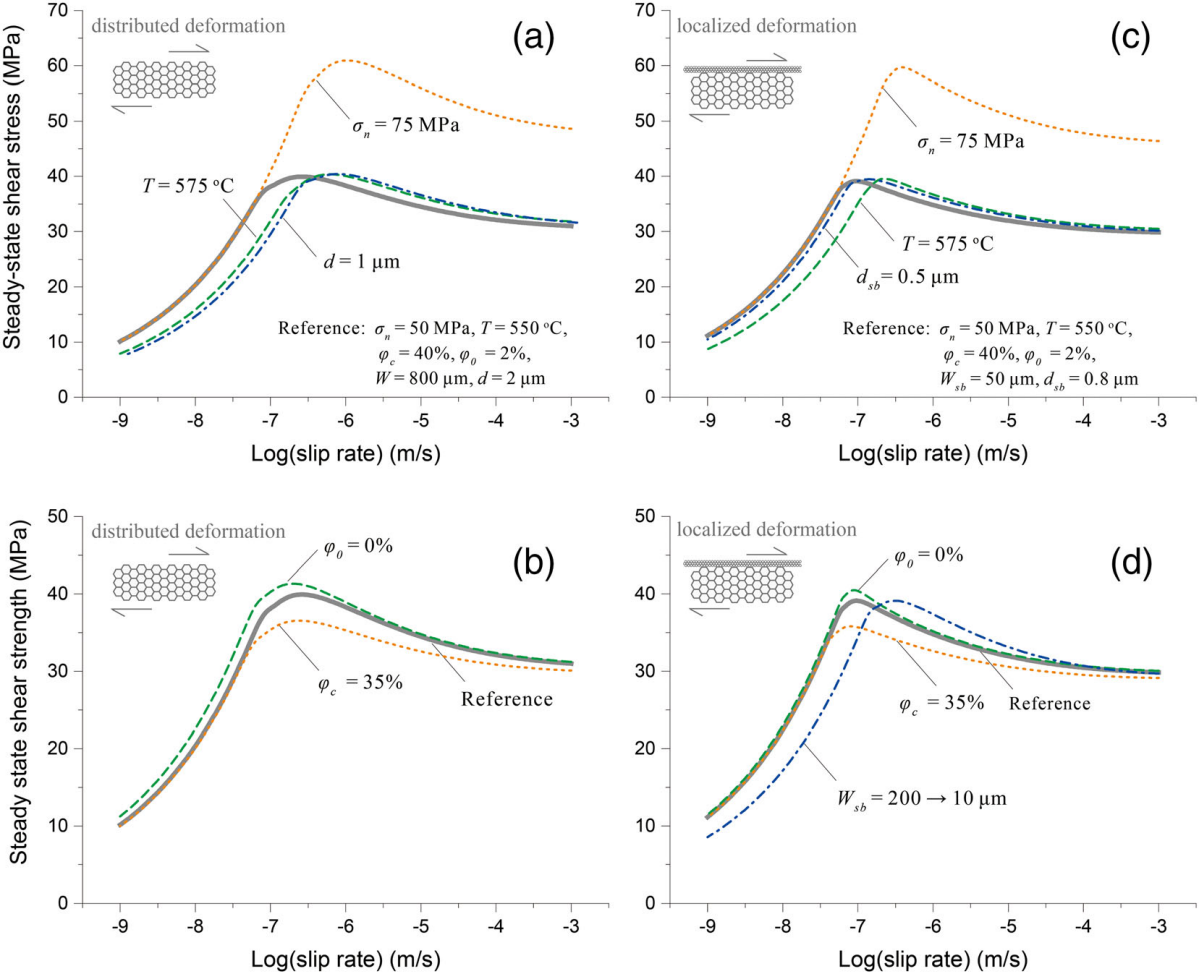
Sensitivity of computed steady‐state friction coefficient to variation in parameter values (*σ*_*n*_, *T*, *d*, *φ*_*c*_, and *φ*_0_, as well as progressively decreasing *W*_*sb*_). Parametric analyses were performed for a wide range of slip rates from 0.001 to 1,000 μm/s, using two fault geometries: (a, b) distributed shear and (c, d) localized slip. For both geometries, the reference cases (thick gray lines) employ the denoted parameter values, and for each other curve, we changed one parameter. All the definitions and values of the parameters are listed in Table 3.

Additional sensitivity analyses, specifically on the effect of varying *σ*_*n*_, *T*, *d*, or *d*_*sb*_, *W*_*sb*_ and *W*_*bulk*_, and *φ*_*c*_ and *φ*_0_ (see Table 3 and supporting information for their definition), consistently showed a *τ‐v* curve characterized by a continuous transition from strong *v‐*strengthening to *v*‐weakening behavior (Figure 9). The critical velocity *v*_*cr*_, which demarcates the transition in the sign of *v*‐dependence, ranges from 0.1 to 0.7 micron/s within the range of parameter values tested. Specifically, an increase in (effective) normal stress (*σ*_*n*_) results in a higher shear strength and an increase in *v*_*cr*_. Increasing the temperature or decreasing the grain size (either *d* or *d*_*sb*_) causes a rightward horizontal translation of the *τ‐v* curve implying a higher *v*_*cr*_‐value. Note that due to the limited thickness of the bulk gouge layer, the grain size (*d*_*bulk*_) has a negligible effect on the shear strength. Lowering *φ*_*c*_ or increasing *φ*_0_ does not change the *τ‐v* profile but leads to a higher peak strength and more pronounced *v*‐weakening in the frictional regime (i.e., for *v* > *v*_*cr*_).

As already shown in Figure 8, a decrease in *W*_*sb*_ causes a leftward horizontal translation of the *τ‐v* curve (see also Figure S6). Here we further investigated the effect of progressive localization, which may have occurred in the fictional regime at *v* > *v*_*cr*_ that showed *v*‐weakening (Beeler et al., 1996). To mimic this, we assumed a log‐linear decrease in *W*_*sb*_ from 200 to 10 μm as *v* increases from the calculated *v*_*cr*_ to 1 mm/s. The predicted *τ‐v* curve displays a higher *v*_*cr*_ and a deeper *v*‐weakening at *v* > *v*_*c*_ (Figure 9d). This may explain why our reference simulation using a constant *W*_*sb*_ predicts a gentler *v*‐weakening than observed in the experiment (see the comparison in Figure S5).

#### 5.2.2 Simulation of Velocity‐Stepping Experiments

We next use the CNS model to investigate the transient shear deformation behavior, as observed in our *v*‐stepping experiments. The experimental setup can be idealized as a spring‐slider system (e.g., Chen & Spiers, 2016). From the initial response upon a perturbation in displacement rate, the apparent stiffness of the loading system measured 55 to 210 GPa/m. Taking a stiffness from this range, the model simulation will sometimes lead to stick‐slips in the frictional regime, especially at relatively low velocities (e.g., for 0.3 μm/s < *v* < 10 μm/s), or when imposing a thin shear band. Although the occurrence of stick‐slip at low *v* is consistent with the findings of Verberne et al. (2015), for calcite gouge sheared under the same *T‐σ*_*n*_ *‐P*_*f*_ conditions as used here, in the present experiments, we consistently observed stable sliding. Therefore, in our model simulations, we employed a stiffness of 500 GPa/m. Other model parameters are set to the same values as used for simulating steady‐state behavior (see Table 3). The initial displacement rate used in the model is set to 0.1 μm/s, beyond which we imposed the same *v*‐stepping sequence as used in the experiments, allowing 0.5 mm of shear displacement in each *v*‐interval. The initial shear stress and porosity were set according to the analytical expressions for steady state (Chen et al., 2017).

**Figure 10:**
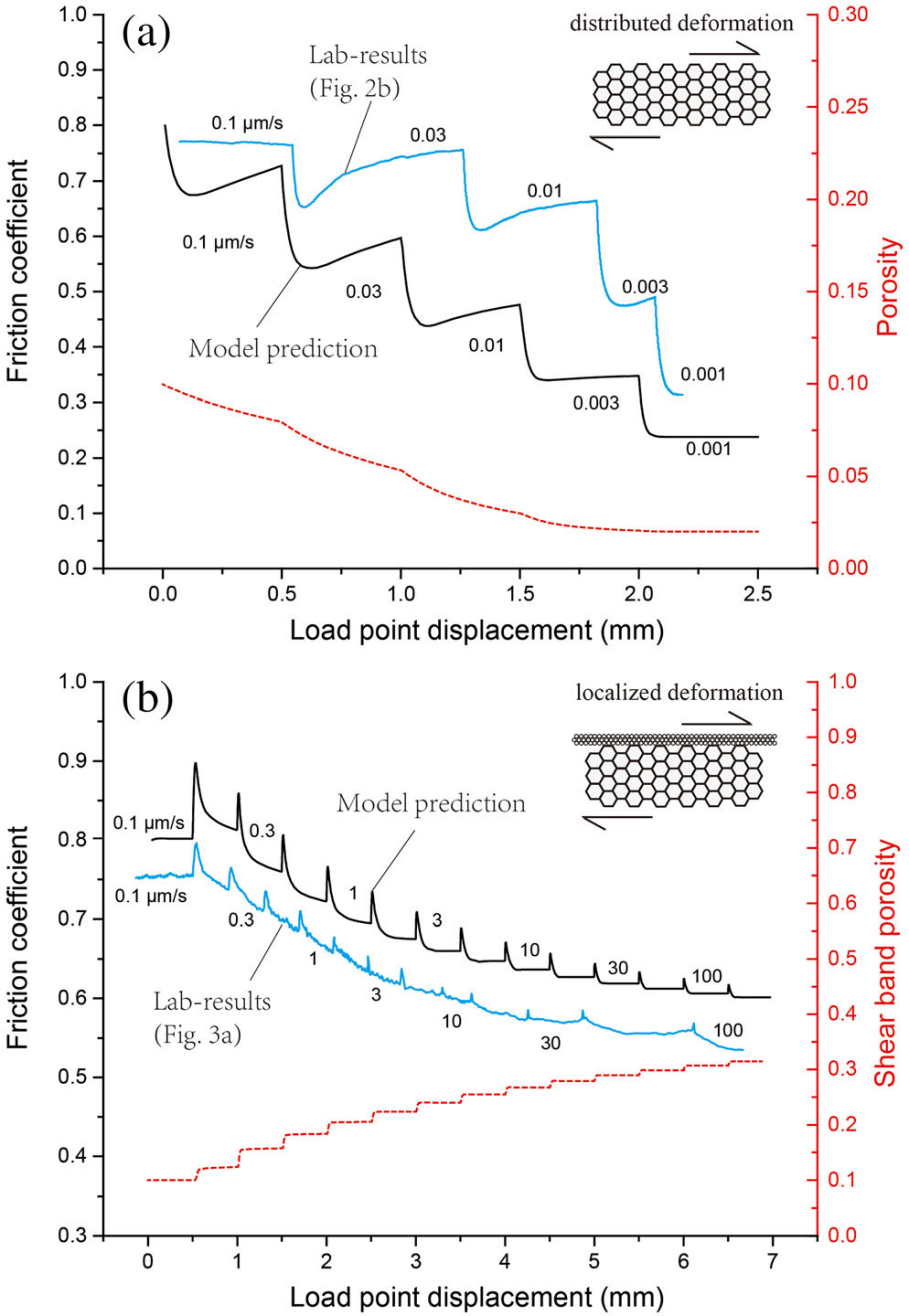
Predicted evolution of friction coefficient and porosity from the CNS model, to simulate (a) the downward and (b) upward *v*‐stepping tests shown in Figures 2 and 3, respectively. The experimental data are added for comparison, with a slight extension of the *x* axis.

**Figure 11:**
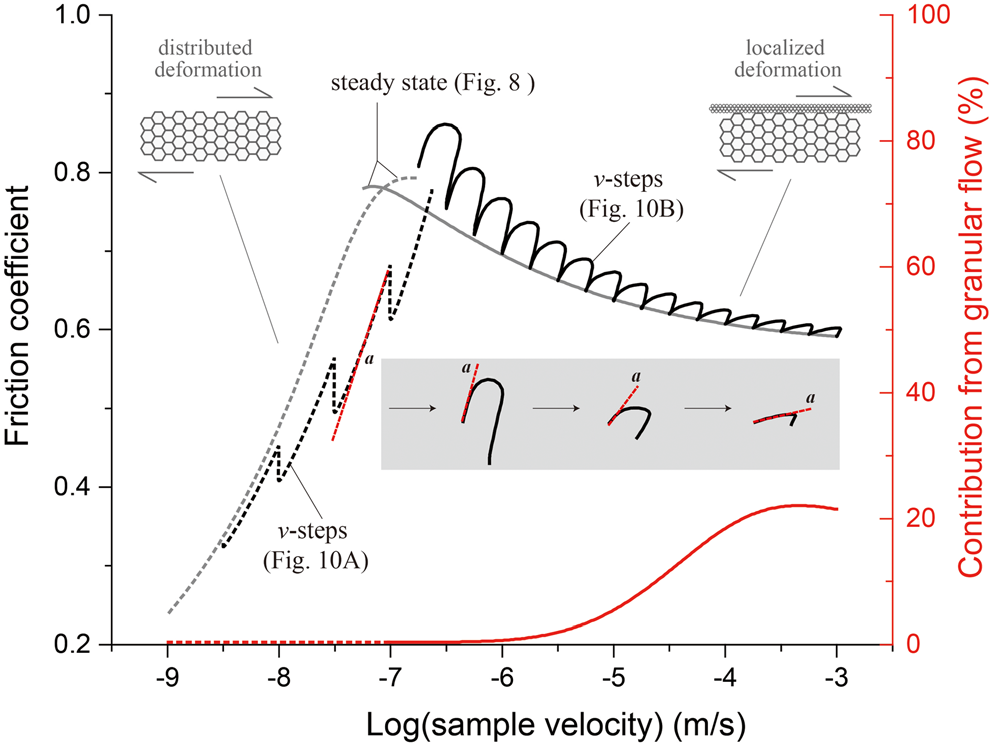
Friction‐velocity phase diagram of the simulated *v*‐steps shown in Figure 10, with the predicted steady‐state shear strength being added for comparison (in gray lines). Results from the friction and flow regimes, with distributed and localized deformation, are plotted in solid and dashed black lines, respectively. The red lines give the relative contribution from granular slip to the shear deformation. The gray inset illustrates the systematic decrease in the direct effect (or *a*‐value) with increasing velocity.

The model output alongside the experimental data are plotted as friction coefficient and porosity versus displacement in Figure 10. For experiments conducted using *v* < *v*_*cr*_, the predicted friction response shows a sharp drop followed by gradual re‐strengthening for the first three steps (*v* ≤ 0.01 μm/s), comparing favorably with the experimental data (Figure 10a). For each displacement rate tested, the model predicts continued compaction with increasing displacement. For *v* ≥ 0.003 μm/s, when the porosity reaches the background level of *φ*_0_, the shear strength shows a monotonic decay, without re‐strengthening. A plot of friction versus sample (or particle) velocity (i.e., *μ* − *v*_*s*_), termed a phase diagram by Gu et al. (1984), shows that the model simulation of downward *v*‐steps defines a curve which is parallel to the interpolated experimental data (Figure 11), with a gap that decreases with increasing slip rate.

The model output simulating the response in shear strength upon a step in displacement rate in *v*‐step tests using *v* > 0.1 μm/s is strikingly consistent with the experimental data (Figures 10b and 11). First, all simulated upward *v*‐steps showed a classical, RSF‐type frictional response, constituting *v*‐weakening. Second, when using the same magnitude *v*‐steps (1.75‐fold), the difference in *μ*_*ss*_ before and after a *v*‐step becomes less as the post‐step *v* increases, implying an increase of the steady‐state frictional rate dependence (i.e., the *a* − *b* value becomes less negative) with increasing *v*. Third, the model output as well as the experimental data show a systematic decrease in the direct effect (i.e., the *a*‐value) with increasing slip rate (see also the inset of Figure 11). The same trend also describes the characteristic slip distance (i.e., the *D*_*c*_‐value). Lastly, for *v* ≤ 3 μm/s, friction‐displacement curves representing the model as well as the experimental data do not reach steady state within 0.5 mm of displacement, whereas for *v* > 10 μm/s, they do. Interestingly, the CNS model produces a long evolution distance for *v*‐steps with *v* just above *v*_*cr*_, which is effectively similar to typical slip‐weakening behavior (Figure 10b).

The direct effect, defined as *a*  =  *dμ/d* (ln*v*), can be directly measured as the slope of the instantaneous response in the *μ*‐*v*_*s*_ phase diagram multiplied by ln (Chen & Spiers, 2016) (see the inset of Figure 11). We found that the direct effect continuously evolves from a flow‐like process at low *v* to granular flow at high *v*. Specifically, for low velocity (*v*  <  *v*_*cr*_), it measures as *a*  =  *a*_*flow*_  =  *μ/n* where *n* is the stress exponent (Equation 2), while at high velocity (*v*  >  *v*_*cr*_), its value gradually decreases from *a*_*flow*_ to $a_{\tilde{\mu }}$ which in the limit approaches the direct effect defined in the RSF model (see Chen & Spiers, 2016). To further specify this, we investigate the relative contribution to shear strain accommodation of plastic flow versus granular flow, at steady state (see the red curves in Figure 11). In the flow regime (*v*  <  *v*_*cr*_), shear deformation is fully accommodated by plastic flow, except that created small increment of porosity starts to play a role at *v*  >  *v*_*dil*_. As slip rate increases, granular flow plays an increasingly important role, ultimately accounting for up to 22% of the total shear strain rate. Their relative contribution determines the *a*‐value, that is, *a* $=\eta /a_{\tilde{\mu }}+\left(1-\eta \right)/a_{\text{flow}}\;$, where *η* $={\dot{\gamma }}_{\mathrm{gr}}/\left({\dot{\gamma }}_{\mathrm{gr}}+{\dot{\gamma }}_{\mathrm{pl}}\right)$ is the relative contribution from granular flow to the shear deformation.

## 6 Discussion

### 6.1 Flow‐to‐Friction Transition and “Semi‐Brittle Flow” of Carbonates at 550°C

In this study, we reported ring‐shear experiments on layers of wet simulated calcite fault gouge sheared at 550°C and 50 MPa effective normal stress conditions, at sliding velocities ranging from 0.001 to 300 μm/s. A plot of steady‐state shear strength against sliding velocity (*v*) showed a transition with increasing *v* from *v*‐strengthening to *v*‐weakening, characterized by a peak shear strength at a critical velocity *v*_*cr*_ = 0.1 μm/s (Figure 4). Samples deformed at *v* < 0.1 μm/s are characterized by a dense, near‐homogeneously deformed microstructure (except slight grain elongation, Figure 5a), compared with localized deformation in samples deformed at *v* > 0.1 μm/s. Our mechanical and microstructural findings are consistent with a transition with increasing slip rate from distributed, creep‐controlled flow to localized, frictional slip beyond *v* ≈ 0.1 μm/s. In the low‐*v* flow regime, deformation is accommodated by compactive, plastic creep processes involving the entire width of the gouge layer. Toward higher slip rates (*v* > 0.1 μm/s), and in the case of localized slip, shear deformation by granular flow plays an increasingly important role. Despite the dramatic differences in the mechanical and microstructural characteristics between the “slow” and the “fast” shear deformation regimes, the creep mechanisms occurring between the grains may be modeled using an empirical constitutive law which represents a mixture of diffusion and dislocation creep.

The stress sensitivity or *n*‐value determined for deformation in the flow regime showed an increase with increasing *v*, from 2.5–4.2 (*v* ≤ 0.01 μm/s or $\dot{\gamma }$ ≤ 1.25 × 10^−6^ s^−1^) to 8.8–87 (*v* → 0.1 μm/s, or $\dot{\gamma }$ → 1.25 × 10^−5^ s^−1^) (Figure 7). An increase of the *n*‐value from 2.1 to 4.2 with increasing strain rate was reported from compression tests on dense calcite aggregates at 500–600°C, by Bruhn et al. (1999). From the present post‐mortem microstructures (Figure 5; see also Verberne et al., 2017) and thickness measurements (Figure S4), as well as the microphysical analysis of steady‐state behavior (Figure 8), we posit that the change in *n*‐value (or slope in strain rate‐stress curve) is caused by porosity development, or cavitation, at grain boundaries. Based on our microphysical model simulations (Figure 8), intergranular cavitation is expected to become noticeable in the gouge shear mechanical properties when the sliding velocity overcomes the dilatancy velocity *v*_*dil*_. With further increasing *v*, cavitation continues until the critical velocity *v*_*cr*_ is reached, which demarcates the flow‐to‐friction transition (Figure 4) accompanied by the change from potentially stable to unstable slip. Relatively high *n*‐values and the development of porosity have also been observed in creep‐type experiments on synthetic feldspar and granitoid rocks, conducted under conditions simulating the BDT (Delle Piane et al., 2009; Pec et al., 2016; Rybacki et al., 2008), and are often referred to as “semi‐brittle flow” behavior (Fredrich et al., 1989; Nicolas et al., 2017). Besides the dependence on velocity, the semi‐brittle nature can be verified from the emergence of normal stress independence of shear strength (Verberne et al., 2017). As shown by the parametric analyses, at a higher normal stress, it is feasible to have continued deformation by purely plastic flow (without dilatancy) at elevated strain rates and therefore a higher *v*_*dil*_‐value (Figures 9a and 9c).

In the semi‐brittle shear deformation regime, the transient response to a sudden drop in loading velocity displays a sharp drop in shear stress followed by a gradual rise to a new steady state (Figure 2). This is like that expected from a frictional response. Such transient behavior has been observed in simulated halite(‐mica) gouges sheared at room temperature and slow slip rates (0.03–0.1 μm/s), as a precursor to a transition from *v*‐strengthening to *v*‐weakening (Niemeijer & Spiers, 2005). From our modeling results, it appears as if deformation in the semi‐brittle regime remains nearly fully plastic (i.e., >99% contribution, Figure 11); however, porosity development due to cavitation effectively leads to local stress enhancement and hence enhanced creep rates, at grain contacts. In other words, the stress required to accommodate gouge shear deformation by dense plastic flow, at zero or at least very low porosity, is higher than that required to generate porosity and to advance deformation at elevated strain rates. This means that in the semi‐brittle deformation regime, it is energetically more favorable to create porosity than to sustain plastic flow.

### 6.2 Microphysical Modeling and Comparison With Previous Models

Using constraints based on observed or measured properties of sheared calcite fault gouge, the CNS model employed here predicts a flow‐to‐friction transition consistent with the experimental data (Figure 8). The CNS model distinguishes itself from previous constitutive models describing fault deformation in the friction/flow regime such as the two‐mechanism model (Beeler, 2009; Chester, 1994; Estrin & Brechet, 1996; Nakatani, 2001; Noda & Shimamoto, 2010; Reinen et al., 1992; Shimamoto & Noda, 2014), because it is based on lab‐derived observations of microphysical deformation processes.

From the point of view of fault rupture modeling, transient shear deformation behavior is more important than steady state, since the velocities vary greatly during earthquake ruptures and, practically, a seismically active fault is always in some transient stage of the earthquake cycle. We have shown that the CNS model can favorably predict transient responses to *v*‐steps in the friction as well as the flow deformation regime (Figures 10 and 11). In particular, the CNS model predicts RSF‐like behavior within the “semi‐brittle flow” regime, which is consistent with that predicted by the empirical model by Noda and Shimamoto (2010) who fitted a rate‐ and state‐dependent flow law to data from shear experiments on halite conducted at high temperatures. Finally, besides the mechanical behavior, the CNS model predicts an increase in porosity with increasing slip rates across the flow‐to‐friction transition (Figure 10). As for a rupture nucleation process, the model results suggest that it is the development of microporosity (cavitation) that is responsible for the “semi‐brittle flow” behavior prior to the nucleation of an earthquake rupture. Of interest is that the onset of dilatation (cavitation) occurs at a velocity (*v*_*dil*_) before the transition, which, as shall be discussed in the following, has important implications for natural fault deformation at the BDT conditions.

Recently, Aharonov and Scholz (2018) developed a physics‐based constitutive law for rock friction, based on the microphysics of contact creep, using an exponential law, and the coupling with frictional heating (hereafter referred to the A&S model). By considering the temperature and stresses at asperities, which impact the direct rate dependence of friction (or *a*‐value in the framework of RSF theory), their model can lead to local (flash) melting and predict different deformation regimes as a function of slip rate. Significantly, the A&S model predictions are essentially similar to those of the CNS model (Chen et al., 2017; Chen & Niemeijer, 2017). More recently, Aharonov and Scholz (2019) have applied their model to higher temperature and pressure conditions and showed that a BDT with increasing depth is a direct consequence of their model. The common foundation shared by the A&S and CNS models is the limit in net grain contact area, or porosity beyond which shear deformation switches from creep‐controlled flow to normal stress‐dependent (or frictional) sliding. Thus, this porosity or grain contact area limit is crucial for the conditions pertaining to the flow‐to‐friction transition and hence the depth to the BDT.

### 6.3 Limitations and Future Work

A potentially crucial uncertainty which we have not yet considered is to what extent recrystallization (grain growth) occurred during or after shear deformation. As addressed in section 4, the grains in the bulk gouges deformed at *v* < 0.1 μm/s have likely grown with respect to the starting material, in the early hours of the experiments. In the frictional regime (*v* > 0.1 μm/s), one would expect a high porosity (~10–30%) in the active shear band due to the operation of granular flow (Figure 8). However, the post‐mortem microstructure of the shear band occasionally shows polygonal grains with straight boundaries and high‐angle junctions, with a relatively low porosity (<9%, Figure 5c). We infer that the compacted structure could be developed by static recrystallization in the termination stage of the experiments. Based on the observed GSD (*d* = 0.3–1.4 μm, with $\bar{d}$ ~0.8 μm, Figure 5) and a temperature profile upon cooling after the experiment (Figure S7‐A), we can estimate the maximum grain sizes prior to annealing, using the grain growth equation for porous calcite aggregates (e.g., Covey‐Crump, 1997). Assuming initial grain sizes from 0.01 to 1.0 μm, the calculations predict that grain growth mostly occurs within the first 50‐s cooling. For grains with an initial size (*d*_0_) smaller than 0.2 μm, the final sizes after cooling are more or less constant and close to ~0.45 μm (Figure S7‐B), whereas for grains with *d*_0_ exceeding 0.2 μm, the total growth in grain size is limited to 0.25 μm (Figure S7‐C). The predicted minimum grain size of 0.45 μm is roughly consistent with our final observation. Therefore, before terminating shearing, the shear bands may contain a portion of grains that were smaller than the observation and have a systematically smaller mean value ( $\bar{d}$) by ~0.15 μm, with a small portion of grains (~10%) that could be smaller than 100 nm (Figure S7‐D). This variation in the $\bar{d}$‐value does fall in the range of our parametric analyses. However, we cannot rule out that the dynamic grain size during active shear could be even smaller (Verberne et al., 2019). To explore this issue, experiments stopped at short shear displacements, together with ad hoc quenching procedures, are required in the future.

From our experiments as well as the microphysical model, it remains ambiguous as to how semi‐brittle flow, local porosity development, and/or slip instability lead to the formation of a shear band (i.e., spontaneous slip localization and grain size reduction). We speculate that this process may be tied to the development of dilatancy in the semi‐brittle flow regime (*v*_*dil*_ < *v* < *v*_*cr*_). Rationalized from a microscopic point of view, cavities developed at grain boundaries will generate high local stresses, which, added to the already high shear stress around the transition, will cause grain breakage preferably at the cavitated points. As stress continues to build up and more cavities develop, a previously creeping gouge can readily dilate from these cavities, leading to the emergence of strain localization and therefore the incipience of a shear band. In other words, it is the inability of semi‐brittle flow to maintain the contiguity of a creeping gouge layer that leads to local disaggregation and thus the formation of shear band. Previous laboratory and numerical modeling studies also showed, in general, that shear localization occurs due to the presence of local heterogeneities, such as those in porosity and GSD (Hadizadeh et al., 2010, 2015; Nübel & Huang, 2004), which could potentially lead to the ductile‐to‐brittle transition. Of course, a continuous flow‐to‐friction transition, together with the associated microstructural evolution (i.e., localization and grain size reduction), occurs spontaneously in both laboratory and natural shear zones (Platt & Behr, 2011; Wehrens et al., 2017), which is not yet captured by the present model and will be considered in future work.

### 6.4 Implications for Fault Rupture Dynamics Within the BDT Zone

**Figure 12:**
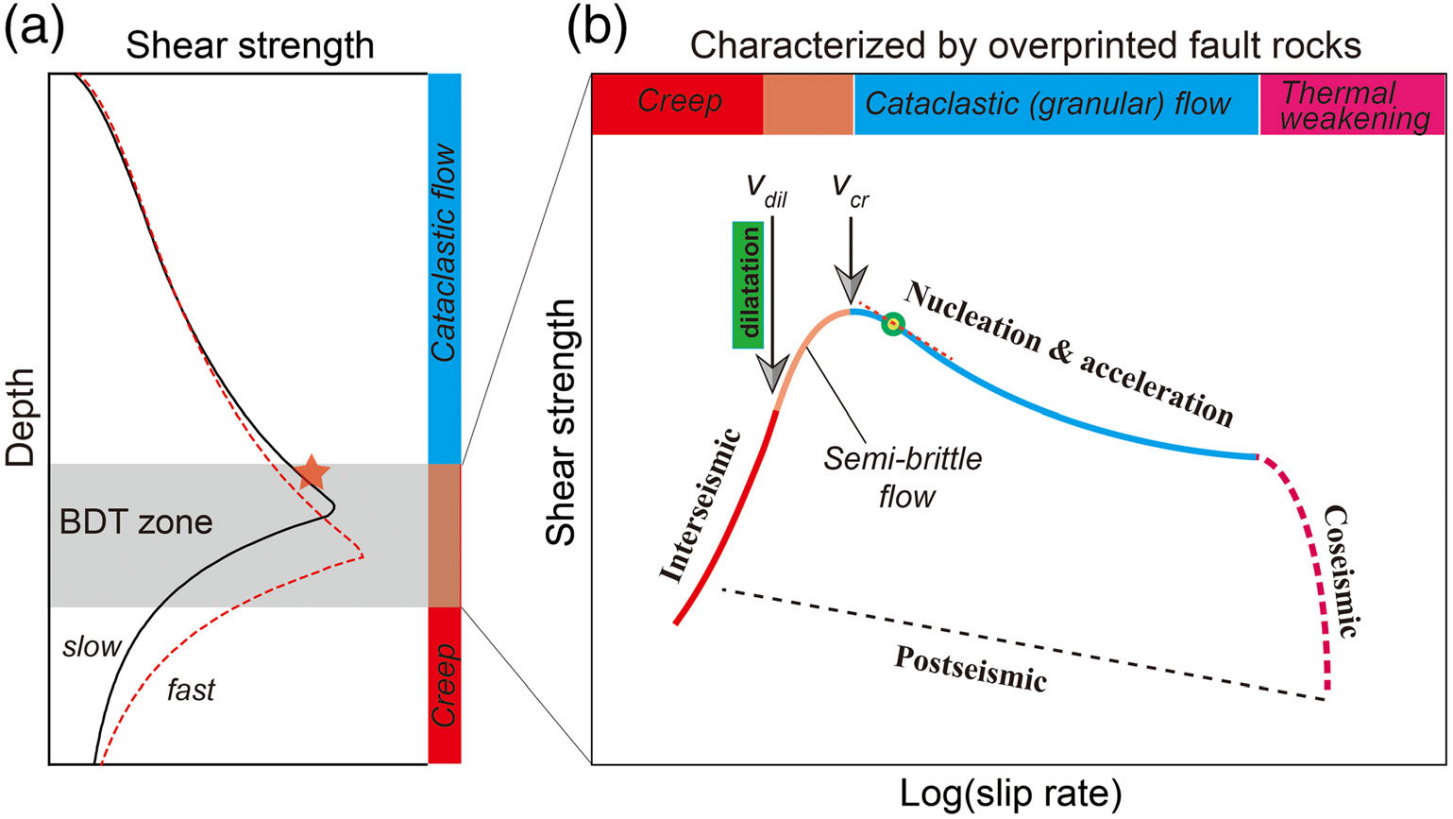
Diagrams illustrating (a) the shear strength‐depth profile of a carbonate fault zone over the crustal depth at fast and slow velocities and (b) the strength‐velocity profile showing the dynamic of an earthquake that nucleates within the brittle‐to‐ductile transition (BDT) zone.

Based on the present experimental and microphysical modeling results, we sketch a diagram showing the shear strength‐depth profile of a carbonate fault (Figure 12a), with the expected fault rupture dynamics in the BDT zone (Figure 12b). For simplicity, the change of shear strength with increasing depth was predicted using the same model (i.e., the case of localized slip), but taking two constant loading velocities (10^−10^ and 10^−9^ m/s) and a geotherm of 25°C/km.

At shallow depths, the shear strength shows a near‐linear increase with increasing depth, representing frictional behavior (Figure 12a). The difference between slow and fast loading velocities indicates a transition from *v*‐strengthening to *v*‐weakening with increasing depth. As depth increases, the peak strength on the profile marks the friction‐to‐flow transition (Figure 12a). As embodied in the CNS model, this transition depth depends not only on the loading velocity but also on fault zone structure such as grain size and shear zone thickness, as well as fault conditions such as the thermal and effective pressure gradients. The variation of this transition depth, resulting from a range of velocities which the fault could potentially experience in an earthquake cycle, then defines the width of the BDT zone (see the gray zone in Figure 12a).

When an earthquake nucleates from a fault patch (or asperity) at the base of the seismogenic zone (see the red star in Figure 12a), which is usually considered to be the upper bound of the BDT zone, it is expected that the fault patch will undergo a transition from stable, ductile flow over a wide shear zone, to unstable, localized frictional slip by cataclastic (granular) flow, involving a wide range of slip rates (see the thick line in Figure 12b). Before the transition, the fault will first show semi‐brittle flow behavior accompanied by the onset of dilatation as described in the earlier sections. Besides the evidence from laboratory experiments, similar mechanical and microstructural characteristics have also been observed in ductile fault rocks collected from natural shear zones exhumed from the aseismic/seismic transition depths (25–35 km, Fusseis & Handy, 2008; Fusseis et al., 2009; Gilgannon et al., 2017; Menegon et al., 2015; Platt et al., 2018; Regenauer‐Lieb, 1999; Shigematsu et al., 2004), sometimes using different terminologies such as “ductile rupture,” “dilatant plasticity,” “dilatant microcracking,” and “creep cavitation.” Our microphysical modeling predicts that “semi‐brittle flow” occurs over a velocity range from the onset of dilatation until the transition to friction (*v*_*dil*_ < *v* < *v*_*cr*_). An important implication is that the mechanical and microstructural features can be taken as indicators of the (aseismic) acceleration stage for a seismogenic fault to produce an instability at higher slip rates.

As the fault accelerates and continues to dilate at *v* > *v*_*cr*_, its shear strength decreases, and an earthquake nucleates (Aharonov & Scholz, 2019). However, as the fault just transitions into the *v*‐weakening regime, the initial minimum nucleation size will be rather large since (*a* − *b*) has only a small negative value. However, as the fault accelerates further, (*a* − *b*) becomes more negative and this size will shrink until it reaches its minimum size at the steepest point in Figure 12b, indicated by the red circle. As the slip area increases beyond the critical nucleation size, the rupture propagates and runaway slip occurs (Scholz, 2002). Finally, as the slip runs away to the coseismic regime (~1 m/s), some thermal weakening mechanisms such as flash heating will start to play a role (Di Toro et al., 2011; Niemeijer et al., 2012), leading to dramatic weakening. For a fault cutting carbonate rocks, one of the candidate mechanisms is grain boundary sliding with accommodation by diffusion creep (De Paola et al., 2015). Implementing this mechanism to explain carbonate dynamic weakening is a natural extension of the present model (i.e., simply using different creep law and with high temperature generated by frictional heating) and is in progress.

Finally, as discussed above, within the BDT zone, the deformation and failure modes might switch between ductile non‐localized plastic flow and brittle‐localized patterns within the time frame of earthquake cycles. The resultant fault rocks will be characterized by repeated overprinting of different deformation processes, specifically interseismic mylonitization, subseismic cataclasis and localization, and coseismic melting or superplasticity. These include pseudotachylyte overprinted with mylonitic deformation, mylonitized cataclasite, and cataclasite containing mylonite clasts (e.g., Fagereng, 2011; Frost et al., 2011; Fusseis et al., 2009; Rowe & Griffith, 2015; Takagi et al., 2000; Toy et al., 2011; Wehrens et al., 2016, 2017; Wintsch & Yeh, 2013). It is noteworthy that what is more commonly seen in outcrops are different layers of fault rocks coexisting across the fault zone (mylonite, cataclasite, pseudotachylyte, and fault gouge), which might form separately in different scenarios (e.g., along with the exhumation of the fault toward the surface).

## 7 Conclusions

In this study, we performed constant‐velocity and velocity‐stepping tests on layers of simulated calcite fault gouge at 550°C, 50 MPa effective normal stress, and 100 MPa fluid pressure conditions, with slip rates covering almost 6 orders of magnitude (0.001–300 μm/s). The shear strength observed at these velocities shows a flow‐to‐friction transition within increasing slip rates, with a critical velocity (*v*_*cr*_) of 0.1 μm/s.

Distinct microstructures were displayed in the two regimes. In the flow regime (*v* < 0.1 μm/s), the gouge is well compacted, displaying a progressive homogeneous texture as slip rate decreases, while in the frictional regime (*v* ≥ 0.1 μm/s), a localized shear band was developed. A stress sensitivity with approximate *n*‐values of 2.5–8.8 was recognized for the flow regime, which, in combination with the characteristic microstructure (i.e., compacted, polygonal grains with high junction angles, some with subtle elongation) and CPO pattern observed, suggests deformation by a mixture of dislocation and diffusion creep. The same creep mechanism was inferred to also occur in the friction regime but is expected to accommodate only a part of the shear deformation, with the rest accommodated by granular flow which generates porosity and in turn enhances local stress and creep rate.

Incorporating the microstructures and inferred creep mechanisms, the microphysical model (CNS model) reproduces the steady‐state shear strength profile showing the transition from flow to friction with increasing slip rate, as well as the transient flow/friction behavior in the flow/friction regime. In the frictional regime (*v* > *v*_*cr*_), the model predicts typical *v*‐weakening behavior; as velocity increases, there is a systematic decrease in the absolute value of (*a* − *b*) and the *a*‐ and *D*_*c*_‐values. The flow regime can be divided into two subregimes, separating from a velocity for the onset of dilatation (*v*_*dil*_). At *v* < *v*_*dil*_, the fault deforms by pure plastic flow following a power law, while at *v* > *v*_*dil*_, the fault deforms by “semi‐brittle flow,” characterized by high stress sensitivity and a transient behavior similar to the RSF frictional behavior. All the predictions are generally consistent with the observations from experiments.

Implications for the dynamics of earthquake ruptures at the BDT zone are made based on the results from present experiments and microphysical model. In particular, our results show that the semi‐brittle flow is occurring at velocities ranging from *v*_*dil*_ to *v*_*cr*_, which is linked to the opening of transient microporosity (or cavitation).

## Supporting information



Supporting Information S1

## Data Availability

Experimental raw data and microphysical models with input data are all freely available online (https://doi.org/10.4121/uuid:63a7dbde‐e223‐43ad‐b184‐bc7f111f883c).
