# Supplementary material for: Flow‐to‐Friction Transition in Simulated Calcite Gouge: Experiments and Microphysical Modeling
Source: J Geophys Res Solid Earth. 2020 Nov 18;125(11):e2020JB019970. doi: 10.1029/2020JB019970 (PMC7757227; doi:10.1029/2020JB019970)
Supplement: Supplementary file 1 — Supporting Information S1 [file JGRB-125-e2020JB019970-s001.docx]

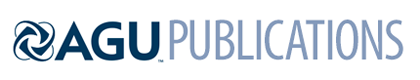


*Journal of Geophysical Research*

Supporting Information for

**Reply to Comment by Peterie et al. on “Accelerated Fill-up of the Arbuckle Group Aquifer and Links to U.S. Midcontinent Seismicity”**

Esmail Ansari^1^ and Tandis S. Bidgoli^2*^

^1^1030 Jana Dr, Lawrence, KS, 66049

^2^Department of Geological Sciences, University of Missouri, Columbia, Missouri 65211, USA

^*^Corresponding author: bidgolit@missouri.edu

**Contents of this file**

Text S1 to S2

Figures S1 to S4

Table S1

**Additional Supporting Information (Files uploaded separately)**

Caption for Dataset S1

**Introduction**

This Supporting Information includes supplementary explanations of referenced reservoir engineering concepts and associated figures, tables, and data captions.

**Text S1.**

**Formation pressure and skin**

Figure S1 schematically shows bottomhole pressure (which is the injection pressure provided by the column of fluid in Class I wells operating by gravity drainage), the average formation pressure (P*), and pressure drop due to skin, $\Delta P_{skin}$ (an area of low permeability). Fall-off tests are used to measure average formation pressure in the reservoir, which is different from bottomhole pressure. Near wellbore formation damage and permeability change (e.g. due to solids plugging pores) can cause additional pressure drop ($\Delta P_{skin})$ and is known as skin. For fluid to flow into the formation, we should have $P_{inj} (or P_{BHP})>\Delta P_{skin}+P^{*}$. The skin effect creates a difference between P* and bottomhole pressure for the same amount of flow (Q) because it reduces permeability in the Darcy equation $Q=\frac{k A}{\mu}\frac{\Delta P}{\Delta x}$. All Class I wells have reported skin factors and pressure drop due to skin. Figure S1b summarizes the compiled skin factors reported for Class I wells. The goal of a fall-off test is to calculate skin factor (i.e. representing pressure drop due to skin), average formation pressure (P*), and formation permeability. See supplementary Materials of Ansari et al. (2019) for how to calculate P* using Horner analysis and detailed formulation for P*.

**Text S2.**

**Superposition principle**

The superposition principle, combined with analytical models, provides an elegant framework for studying the effects of wells and their varying injection rate on the pressure. Temporal superposition accounts for the effect of varying injection rate on the pore pressure (Figure S2). In time-varying rate superposition, the initial rate $q_{1}$ acts over the entire period $t_{n}$ and at each time a new well is opened in the same location with the new rate (for example $q_{2}$) adjusted by the previous rate and acting over the entire remaining time period (i.e. $t_{n}-t_{2})$ and so on as is demonstrated in Table S1 and Figure S2 (Dake, 2008). Spatial superposition accounts for the pressure and stress contribution of thousands of wells on a single point in space and simply adds the pressure contribution by each well.


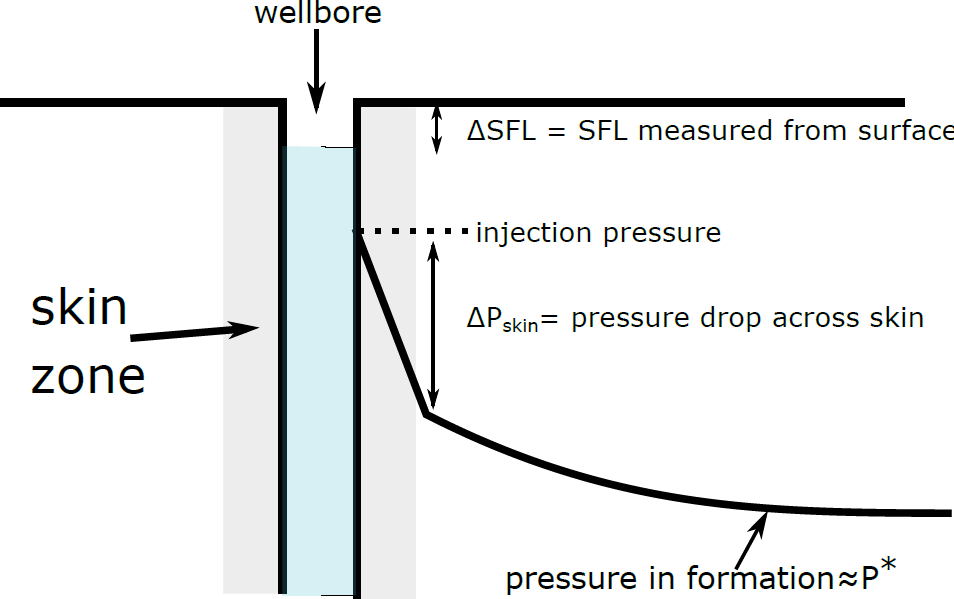

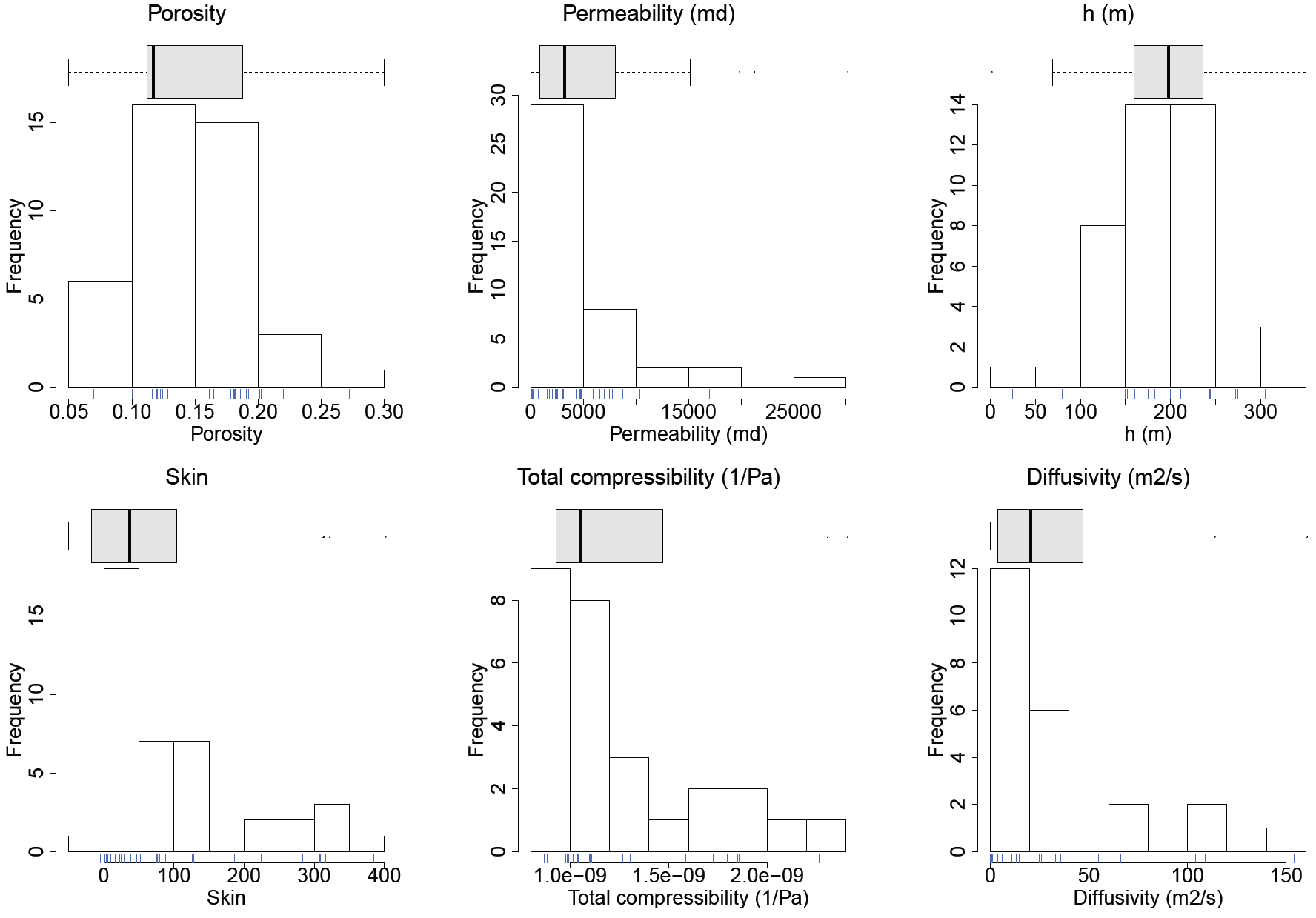


b)

a)

Figure S1. a) Illustration showing the injection pressure, pressure in the formation ($\boldsymbol{\approx}$P*), and pressure drop due to skin effect measured by fall-off tests. b) Summary of skin factors reported for Class I wells inducing$\boldsymbol{large \Delta}\mathbf{P}_{\mathbf{skin}}$.


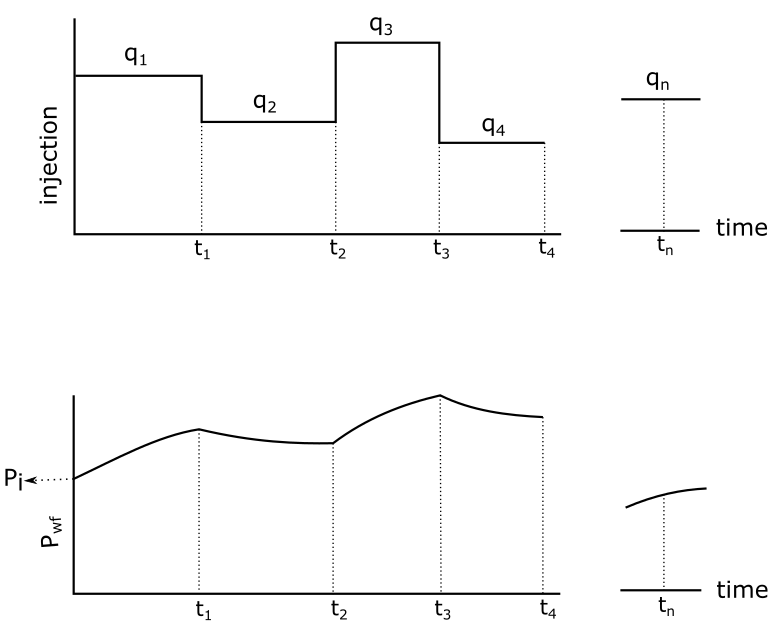


**Figure S2.** Diagram illustrating temporal superposition principle.


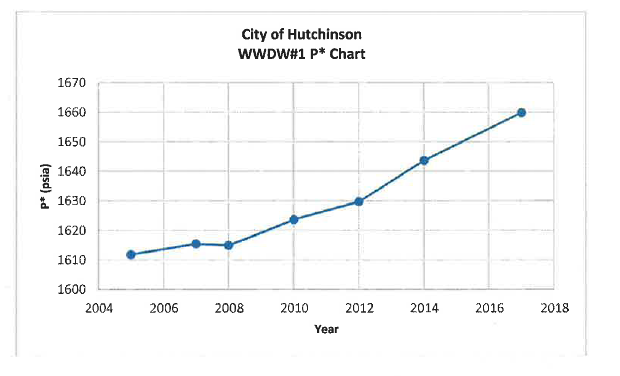


Figure S3. Pressure for well KS-01-155-008 located in Reno County offering more data than Ansari et. al. (2019). This well has not had any change in gauge depth and does not show any pressure plume arrival (Figure directly from Class I reports).


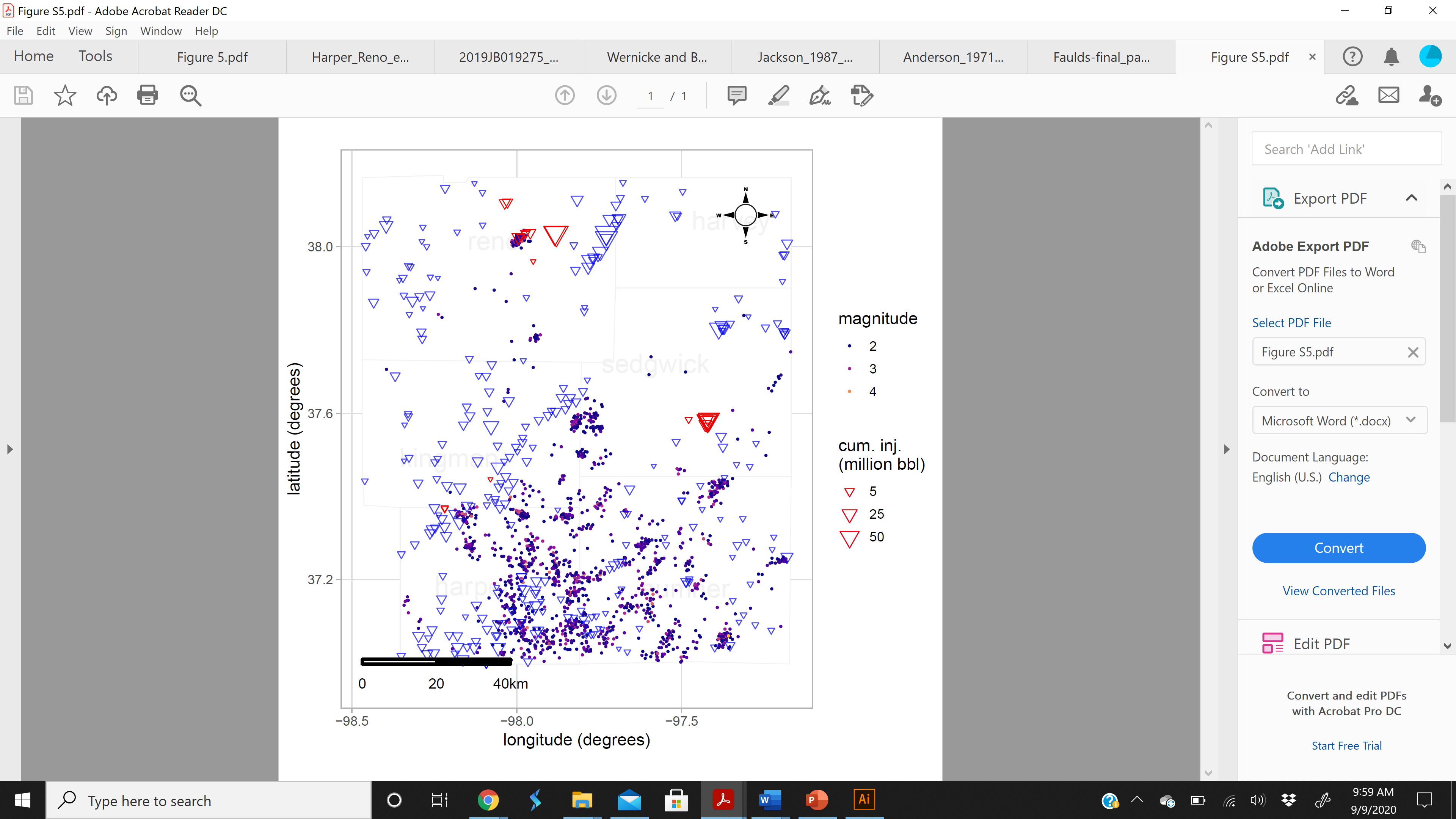


b)

Figure S4. Earthquakes from January 1, 2016 through September 9, 2020 reported by the Kansas Geological Survey’s Seismometer Network. The network has been in operation since the end of 2015 and includes state funded seismic stations and others that are privately funded through a consortium of Class I industrial well operators (<http://www.kgs.ku.edu/Geophysics/CSTS/about.html>). Although the earthquake locations from this network are available, the waveform data are not public and thus, locations from this network cannot be verified. Nonetheless, the pattern of earthquakes is similar to those from the USGS catalog. While far-field pressure diffusion is a possible (though unlikely) explanation for the occurrence of earthquakes in Reno County, a simpler explanation is that these earthquakes are related to high-rate Class I injection wells (red triangles) in the county, which have been injecting at high rates for more than 10 years and are in close proximity (25-30 km) of these events. Blue triangles are Class II saltwater disposal wells.

Table S1. Superposition principle for time-varying rate

| $q_{1}$ | Acting for time | $t_{n}$ |
| --- | --- | --- |
| $+\left( q_{2}-q_{1} \right)$ | “ | $(t_{n}-t_{1})$ |
| $+\left( q_{3}-q_{2} \right)$ | “ | $(t_{n}-t_{2})$ |
| $\ldots$ | … | … |
| $+\left( q_{n}-q_{n-1} \right)$ | “ | $(t_{n}-t_{n-1})$ |

Data S1. **(separate file)** Class I well gauge depth data with annotations by reply authors.
